# Functional Neuroligin-2-MDGA1 interactions differentially regulate synaptic GABA_A_Rs and cytosolic gephyrin aggregation

**DOI:** 10.1038/s42003-024-06789-z

**Published:** 2024-09-17

**Authors:** Tommaso Zeppillo, Heba Ali, Sowbarnika Ravichandran, Tamara C. Ritter, Sally Wenger, Francisco J. López-Murcia, Erinn Gideons, Janetti Signorelli, Michael J. Schmeisser, Jens Wiltfang, JeongSeop Rhee, Nils Brose, Holger Taschenberger, Dilja Krueger-Burg

**Affiliations:** 1 Department of Molecular Neurobiology, Max Planck Institute for Multidisciplinary Sciences, 37075 Göttingen, Germany; 2 Institute of Anatomy, University Medical Center of the Johannes Gutenberg-University Mainz, 55128 Mainz, Germany; 3 Göttingen Graduate School for Neurosciences, Biophysics, and Molecular Biosciences (GGNB), Georg-August-University Göttingen, 37077 Göttingen, Germany; 4 Focus Program Translational Neurosciences (FTN), University Medical Center of the Johannes Gutenberg-University Mainz, 55128 Mainz, Germany; 5 Departamento Biomedico, Facultad de Ciencias de la Salud, Universidad de Antofagasta, 1240000 Antofagasta, Chile; 6 Department of Psychiatry and Psychotherapy, University Medical Center of the Georg-August-University Göttingen Mainz, 37075 Göttingen, Germany; 7 German Center for Neurodegenerative Diseases (DZNE), 37075 Goettingen, Germany; 8 Neurosciences and Signaling Group, Institute of Biomedicine (iBiMED), Department of Medical Sciences, University of Aveiro, Aveiro, Portugal; 9 Present Address: Department of Pathology and Experimental Therapy, Institute of Neurosciences, University of Barcelona, and Bellvitge Biomedical Research Institute (IDIBELL), 08907 L’Hospitalet de Llobregat, Barcelona, Spain

**Keywords:** Synaptic transmission, Diseases of the nervous system, Molecular neuroscience

## Abstract

Neuroligin-2 (Nlgn2) is a key synaptic adhesion protein at virtually all GABAergic synapses, which recruits GABA_A_Rs by promoting assembly of the postsynaptic gephyrin scaffold. Intriguingly, loss of Nlgn2 differentially affects subsets of GABAergic synapses, indicating that synapse-specific interactors and redundancies define its function, but the nature of these interactions remain poorly understood. Here we investigated how Nlgn2 function in hippocampal area CA1 is modulated by two proposed interaction partners, MDGA1 and MDGA2. We show that loss of MDGA1 expression, but not heterozygous deletion of MDGA2, ameliorates the abnormal cytosolic gephyrin aggregation, the reduction in inhibitory synaptic transmission and the exacerbated anxiety-related behaviour characterizing Nlgn2 knockout (KO) mice. Additionally, combined Nlgn2 and MDGA1 deletion causes an exacerbated layer-specific loss of gephyrin puncta. Given that both Nlgn2 and the MDGA1 have been correlated with many psychiatric disorders, our data support the notion that cytosolic gephyrin aggregation may represent an interesting target for novel therapeutic strategies.

## Abstract (web summary)

Analysis of inhibitory synapses in mouse hippocampus uncovers functional interactions between postsynaptic organizer proteins Neuroligin-2 and MDGA1 in regulating gephyrin aggregation, GABAergic synaptic transmission, and behavior.

## Introduction

Information flow in the brain is critically shaped by synapses, specialized contact sites between neurons that are not mere passive relays but actively contribute to information processing and network output. Accordingly, the molecular machinery that mediates synaptic connectivity and neurotransmission plays a central role in regulating cognition and behavior, and alterations in this machinery, e.g. due to genetic or environmental causes, feature prominently in the pathophysiology of psychiatric and neurodevelopmental disorders^1–5^. Therefore, defining the molecular logic that governs the establishment and maintenance of synaptic function represents a problem of utmost importance in the development of new therapeutic strategies for these disorders.

Of particular interest in this respect are alterations in the function of γ-aminobutyric acidergic (GABAergic) inhibitory neurons and synapses, which contribute to a plethora of computational network processes in health and disease. Fast GABAergic neurotransmission is mediated GABA type-A receptors (GABA_A_Rs), whose postsynaptic localization and function are regulated by a complex machinery of scaffolding and cell adhesion proteins^5,6^. A key component of this GABAergic postsynaptic protein machinery is the cell adhesion protein Neuroligin-2 (Nlgn2), which binds trans-synaptically to presynaptic neurexins (Nrxns), and intracellularly to gephyrin and collybistin to establish and regulate GABAergic synaptic transmission^7,8^. Accordingly, deletion of Nlgn2 in mice results in a loss of gephyrin and GABA_A_R subunits from postsynaptic sites, and in a reduction in the frequency and/or amplitude of miniature inhibitory postsynaptic currents (mIPSCs)^7,9–14^.

An intriguing and as yet unexplained feature of Nlgn2 function at GABAergic synapses is its apparent synapse subtype specificity. Despite being present at virtually all inhibitory synapses in the brain, as assessed by immunohistochemical co-localization with gephyrin, deletion of Nlgn2 in mice selectively affects a distinct subset of GABAergic synapses, most notably perisomatic, likely parvalbumin-positive synapses onto principal neurons^8–11,13^. Given that GABAergic neurons are highly diverse, with different subtypes playing distinct roles in shaping network function and behavioral output^15–19^, understanding the molecular mechanisms underlying the striking synapse-type specificity of Nlgn2 function is key for elucidating its role in network function and information processing in health and disease. A likely explanation lies in the differential interaction of Nlgn2 with other synaptic adhesion proteins that differentially localize to GABAergic synapse subtypes. In recent years, multiple new organizer proteins were identified at GABAergic synapses, but the mechanisms by which these might contribute to the differential formation and function of GABAergic synapse subtypes are still largely unknown^5,20^.

**Fig. 1:**
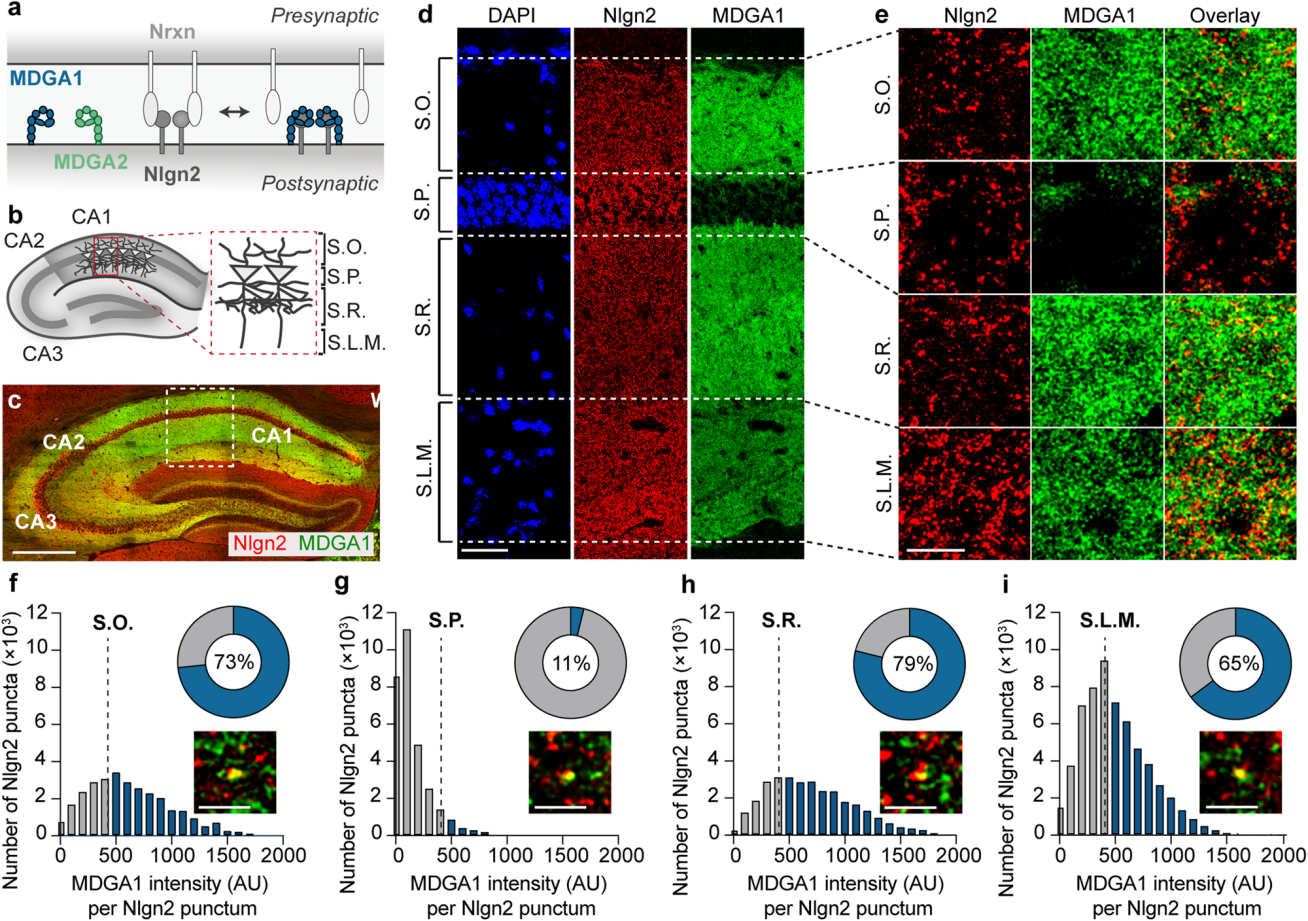
Nlgn2 and MDGA1 colocalization in hippocampal CA1 layers in WT mice. **a** Simplified model for the putative interaction between MDGAs and Nlgn2 in the synaptic cleft. Note that MDGA1 and MDGA2 have both been reported to bind to Nlgn2, but not at the same time. **b** Schematic representation of the dorsal hippocampus showing the layers of area CA1 in which images were acquired: Stratum oriens (S.O.), Stratum pyramidale (S.P.), Stratum radiatum (S.P.) and Stratum lacunosum-moleculare (S.L.M). **c** Photomicrograph showing a low magnification overview of the dorsal hippocampus of a WT mouse labelled with antibodies against Nlgn2 (red) and MDGA1 (green). Scale bar 500 µm. **d** Photomicrographs showing an overview of hippocampal area CA1 of a WT mouse labelled with DAPI (blue) and with antibodies against Nlgn2 (red) and MDGA1 (green). Scale bar 50 µm. **e** High magnification photomicrographs obtained from each layer showing Nlgn2 (red) and MDGA1 (green) labeling. Scale bar 5 µm. **f**–**i** Histograms showing the frequency distribution of MDGA1 fluorescence intensity in arbitrary units) within Nlgn2-labelled puncta in each layer. Bars in blue represent Nlgn2-labeled puncta with above-threshold MDGA1 fluorescence intensity (see Methods section for threshold determination). Doughnut chart insets display the percentage of Nlgn2-labelled puncta with an above-threshold MDGA1 fluorescence intensity (in blue, percentage values are given in the center of the doughnut chart). High magnification photomicrograph insets show examples of MDGA1-colocalized Nlgn2 puncta for each hippocampal layer. Scale bar 2 µm.

Among the newly described postsynaptic interaction partners of Nlgn2 are the MAM-domain containing glycosylphosphatidylinositol anchor (MDGA) family proteins MDGA1 and MDGA2^21,22^. MDGAs were proposed to negatively regulate Nlgn2 function by blocking the interaction between Nlgn2 and its presynaptic partner Nrxn, thereby potentially impairing the assembly of GABAergic synapses^22–30^ (Fig. 1a). Intriguingly, MDGA1 was recently shown to selectively regulate the formation of GABAergic synapses onto distal dendrites, but not proximal dendrites or somata, of hippocampal area CA1 pyramidal neurons through an interaction with presynaptic amyloid precursor protein (APP)^31^. These findings support the notion that MDGAs can contribute to the diversity of GABAergic synapse function, but whether they also differentially modulate Nlgn2 function at different synapse subtypes remains unknown. Moreover, there is substantial controversy over whether MDGA1^25–27^ or MDGA2^32^—or neither^33^—are the most relevant MDGAs for Nlgn2 regulation at GABAergic synapses. We addressed these questions by investigating the function and layer-specific composition of GABAergic synapses in hippocampal area CA1 of Nlgn2/MDGA1 double KO and Nlgn2 KO/MDGA2 heterozygous KO mice. Given that variants of both Nlgn2^7,34–39^ and the MDGAs^21,40–43^ have been linked to schizophrenia, autism spectrum disorders, and other brain disorders, our findings not only have important implications for understanding the basic biology of GABAergic synapses, but may also advance our knowledge on neuropsychiatric disorders linked to dysfunction of GABAergic inhibition.

## Results

### MDGA1 co-localizes with Nlgn2 predominantly in dendritic, but not perisomatic, regions of CA1 pyramidal neurons

To determine how MDGAs may interact differentially with Nlgn2 in the regulation of GABAergic synapse function, we first used immunolabeling to assess the co-localization of MDGAs and Nlgn2 in WT mice. We initially focused on area CA1 in the hippocampus of adult (8–12 week old) mice (Fig. 1a, b) due to the high expression level and known synaptic function of both Nlgn2 and the MDGA proteins in this region^11,25,26,31,33^. Moreover, the layered structure and the precisely defined pattern of connectivity of GABAergic interneurons in area CA1 make this region uniquely suited for the study of the molecular diversity at GABAergic synapses^44^. Since we were unable to identify a good antibody against MDGA2, we focused on the analysis of MDGA1 using a recently reported antibody^33^. Specificity of antibodies against MDGA1 and Nlgn2 was confirmed using Nlgn2/MDGA1 double KO (dKO) mice as a control (Supplementary Fig. 1a, b). A strong expression of both MDGA1 and Nlgn2 was observed in hippocampal regions CA1, CA2 and CA3 (Fig. 1c), consistent with previous reports^33^. Detailed immunolabeling analysis in CA1 revealed a prominent but relatively diffuse distribution of MDGA1 in the Stratum oriens (S.O.), Stratum radiatum (S.R.) and Stratum lacunosum moleculare (S.L.M.), but only low levels in the Stratum pyramidale (S.P.) (Fig. 1d, e and Supplementary Fig. 1c–f, green signal). In contrast, Nlgn2 localization was punctate and strongest in S.P. and S.L.M., and slightly weaker in S.O. and S.R. (Fig. 1d, e and Supplementary Fig. 1c–f, red signal). To investigate the co-localization of MDGA1 with Nlgn2 puncta in the different layers, the intensity of the MDGA1 signal within Nlgn2 puncta was calculated and plotted as a histogram showing the number of Nlgn2 puncta at each MDGA1 signal intensity (Fig. 1f–i). In order to compare the number of Nlgn2 puncta that contained strong MDGA1 immunolabeling in each layer, an empirically determined threshold (signal intensity 10-fold above MDGA1 KO signal) was applied and the percentage of total Nlgn2 puncta with above-threshold MDGA1 immunolabeling was determined (Fig. 1f–i, pie charts). Consistent with the expression intensity of MDGA1, the greatest degree of colocalization with Nlgn2 was observed in S.R. and S.O., less in S.L.M. and very little in S.P. Taken together, these findings indicate that MDGA1 is present primarily in dendritic but not perisomatic compartments of CA1 pyramidal neurons, and that functional interactions with Nlgn2 are likely to take place in both apical and basal dendrites of these neurons.

To further validate our mouse models and exclude a possible compensatory upregulation of MDGA2 expression following MDGA1 loss and vice versa, we quantified MDGA1 and MDGA2 mRNA levels in both MDGA1 KO and MDGA2 Het mice by qRT-PCR (Supplementary Fig. 1g–i). As expected, no MDGA1 mRNA was detected in MDGA1 KO mice, confirming the validity of our KO model, whereas MDGA2 Het mice showed WT-like levels of MDGA1 mRNA (Supplementary Fig. 1g). Similarly, MDGA2 mRNA levels were reduced by approximately 50% in MDGA2 Het mice as expected, but no differences in MDGA2 mRNA levels were observed between WT and MDGA1 KO mice (Supplementary Fig. 1i). Immunoblotting analysis of MDGA1 protein levels also revealed no differences between WT and MDGA2 Het mice (Supplementary Fig. 1j–k). Together, our data indicate that there is no compensatory upregulation of either MDGA family protein following the loss of the other in the mouse models used in the current study.

### MDGA1 differentially co-localizes with Nlgn2 in different subregions of the amygdala

Since our previous work showed that Nlgn2 also plays a key role in mediating synaptic and behavioral functions in the amygdala^9,45,46^, we additionally assessed the colocalization of MDGA1 and Nlgn2 in different subregions of the amygdala of WT mice. While Nlgn2 expression was observed throughout the amygdala (Supplementary Fig. 2a), MDGA1 expression was most pronounced in the ventromedial intercalated cell cluster and the centromedial amygdala (Supplementary Fig. 2b). Accordingly, colocalization of Nlgn2 and MDGA1 was highest in these areas, and lower (but not absent) in the centrolateral and basolateral amygdala (Supplementary Fig. 2c, e, f). These findings indicate that functional interactions between Nlgn2 and MDGA1 may occur across multiple brain regions. However, since it is substantially more challenging to study subcellular connection specificity in a non-layered structure such as the amygdala, we focused the rest of our study on functional Nlgn2-MDGA interactions in the layered structure of hippocampal area CA1.

### MDGA1 and Nlgn2 functionally interact to regulate GABAergic synapses in layer S.R. of hippocampal area CA1

**Fig. 2:**
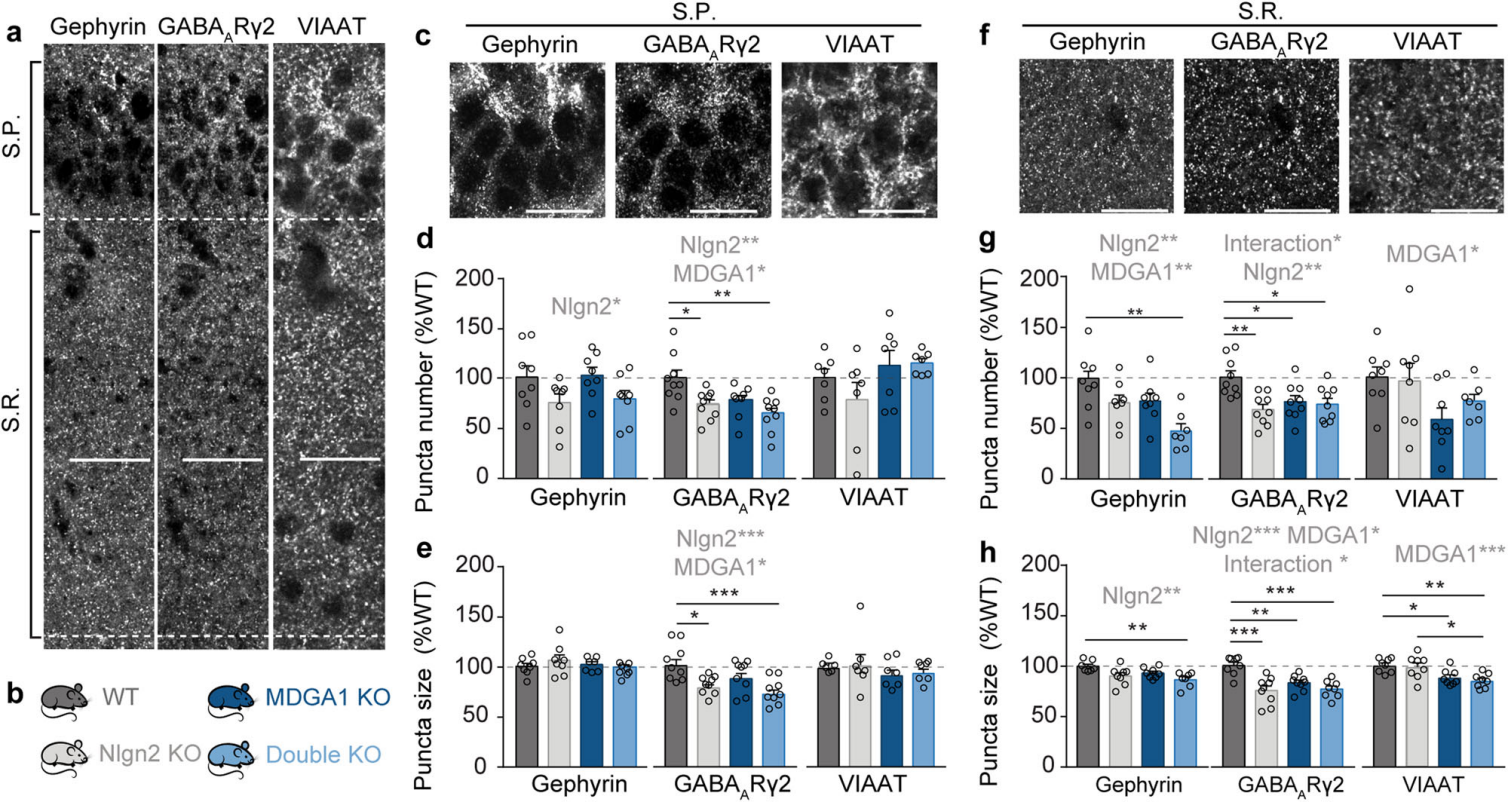
Immunohistochemical characterization of pre- and postsynaptic markers in layers S.P. and S.R. of the adult hippocampal CA1 region. **a** Photomicrographs showing an overview of layers S.P. and S.R. in a WT mouse labelled with antibodies against gephyrin (left), GABA_A_Rγ2 (middle), and VIAAT (right). Scale bar 50 µm. **b** Schematic representation of the four genotypes analyzed in this study. **c** High magnification photomicrographs of layer S.P. in a WT mouse labeled with antibodies against gephyrin (left), GABA_A_Rγ2 (middle), and VIAAT (right). Scale bar 5 µm. **d**, **e** Quantification of the number and size of gephyrin, GABA_A_Rγ2, and VIAAT synaptic puncta in the S.P. **f** High magnification photomicrographs of layer S.R. in a WT mouse labeled with antibodies against gephyrin (left), GABA_A_Rγ2 (middle), and VIAAT (right). Scale bar 5 µm. **g**, **h** Quantification of the number and size of gephyrin, GABA_A_Rγ2, and VIAAT synaptic puncta in the S.R. Statistically significant ANOVA comparisons are marked in gray at the top of panels and are listed in Table 1. For all other ANOVA comparisons, F < 1. Post-hoc analysis (Tukey’s multiple comparison test): *p < 0.05, **p < 0.01, ***p < 0.001. Error bars represent SEM, and each circle represents an experimental animal (*n* = 7–8 for gephyrin; 8–9 for GABA_A_Rγ2, 6–8 for VIAAT).

While the interaction between Nlgn2 and MDGAs has been extensively investigated at the structural level^23,24,30^, its direct functional consequences for GABAergic postsynaptic sites in intact neuronal circuits are poorly understood. To address this question, we investigated the composition of GABAergic postsynapses in hippocampal area CA1 in Nlgn2/MDGA1 dKO mice compared to WT, Nlgn2 KO and MDGA1 KO mice (Fig. 2a, b). We used immunolabeling to identify layer-specific alterations in the inhibitory synapse-specific postsynaptic scaffolding protein gephyrin and the GABA_A_R subunit γ2 (Fig. 2a), which were both shown to be reduced in Nlgn2 KO mice in a synapse subtype-specific manner^11^. Accordingly, we observed a reduction in the number and/or size of gephyrin and GABA_A_Rγ2 puncta in Nlgn2 KO mice which covered layer S.P. (Fig. 2c–e, grey bars, and Table 1), but surprisingly extended to layer S.R. (Fig. 2f–h, grey bars, and Table 1) and, to a lesser degree, to layer S.O. (Supplementary Table 2). In contrast, MDGA1 KO affected gephyrin and GABA_A_Rγ2 staining most prominently in layer S.R. (Fig. 2f–h, dark blue bars, and Table 1), in keeping with our observation that MDGA1 is localized most strongly in this layer (Fig. 1d, e). In particular, MDGA1 KO mice displayed a trend toward a reduction in the number of gephyrin puncta specifically in layer S.R. (Fig. 2f–h, dark blue bars, and Table 1), and this trend was strongly exacerbated in the Nlgn2/MDGA1 dKO mice (Fig. 2f–h, light blue bars, and Table 1). No effect of the MDGA1 KO or its interaction with the Nlgn2 KO was observed in any other layer (Fig. 2c–e and Supplementary Table 2). Similarly, MDGA1 KO most strongly, albeit not exclusively, affected GABA_A_Rγ2 puncta in layer S.R. (Fig. 2f–h, dark blue bars, and Supplementary Table 2). A reduction in both the number and size of GABA_A_Rγ2 puncta was observed in MDGA1 KO mice, which matched the reduction observed in Nlgn2 KO mice and was not further exacerbated in the Nlgn2/MDGA1 dKO mice. More subtle effects of MDGA1 deletion were observed in layers S.P. and S.L.M., with a reduction of the number and size of GABA_A_Rγ2 puncta in layer S.P. and of the number of GABA_A_Rγ2 puncta in layer S.L.M. (Fig. 2c–e, dark blue bars, and Supplementary Table 2, respectively). Together, these findings highlight that MDGA1 deletion most prominently affects GABAergic postsynapses in layer S.R. of hippocampal area CA1, and that functional interactions between the effects of MDGA1 and Nlgn2 are largely restricted to this layer.

**Table 1:** Two-way ANOVA comparisons for Figs. 2–6

Fig.	MDGA1 x Nlgn2 interaction		Main effect of Nlgn2 KO		Main effect of MDGA1 KO	
	*F*-value	*p*-value	*F*-value	*p*-value	*F*-value	*p*-value
**2d** Gephyrin	F_(1,28)_ < 1	0.929	F_(1,28)_ = 7.4	0.011	F_(1,28)_ < 1	0.767
**2d** GABA_A_Rγ2	F_(1,31)_ = 1.2	0.293	F_(1,31)_ = 9.5	0.004	F_(1,31)_ = 6.4	0.017
**2d** VIAAT	F_(1,24)_ < 1	0.335	F_(1,24)_ < 1	0.435	F_(1,24)_ < 1	0.061
**2e** Gephyrin	F_(1,27)_ = 1.4	0.247	F_(1,27)_ < 1	0.641	F_(1,27)_ < 1	0.519
**2e** GABA_A_Rγ2	F_(1,32)_ < 1	0.502	F_(1,32)_ = 15.7	<0.001	F_(1,32)_ = 4.2	0.047
**2e** VIAAT	F_(1,23)_ < 1	0.990	F_(1,23)_ < 1	0.728	F_(1,23)_ = 1.2	0.276
**2g** Gephyrin	F_(1,27)_ < 1	0.607	F_(1,27)_ = 9.5	0.005	F_(1,27)_ = 8.2	0.008
**2g** GABA_A_Rγ2	F_(1,32)_ = 6.4	0.016	F_(1,32)_ = 8.8	0.006	F_(1,32)_ = 2.6	0.116
**2g** VIAAT	F_(1,27)_ < 1	0.383	F_(1,27)_ < 1	0.570	F_(1,27)_ = 6.3	0.019
**2h** Gephyrin	F_(1,26)_ < 1	0.549	F_(1,26)_ = 10.0	0.004	F_(1,26)_ = 4.2	0.052
**2h** GABA_A_Rγ2	F_(1,30)_ = 7.3	0.012	F_(1,30)_ = 20.4	<0.001	F_(1,30)_ = 5.2	0.030
**2h** VIAAT	F_(1,28)_ < 1	0.830	F_(1,28)_ < 1	0.429	F_(1,28)_ = 17.6	<0.001
**3d**	F_(1,26)_ < 1	0.529	F_(1,26)_ = 25.7	<0.001	F_(1,26)_ = 22.7	<0.001
**3e**	F_(1,26)_ < 1	0.296	F_(1,26)_ = 17.8	<0.001	F_(1,26)_ = 20.7	<0.001
**4d**	F_(1,73)_ = 3.6	0.064	F_(1,73)_ = 5.8	0.025	F_(1,73)_ = 13,7	<0.001
**4f**	F_(1, 69)_ = 7.1	0.01	F_(1, 69)_ = 43.2	<0.0001	F(1, 69) = 4.2	0.044
**4h**	F_(1, 54)_ = 3.9	0.055	F_(1, 54)_ = 21.7	<0.0001	F_(1, 54_) = 0.7	0.422
**4l**	F_(1, 68)_ = 12.1	0.001	F_(1, 68)_ = 55.0	<0.0001	F_(1, 68)_ = 0.1	0.711
**4o** mIPSC (All)	F _(1, 54)_ = 9.2	0.004	F _(1, 54)_ = 57.2	<0.0001	F _(1, 54)_ = 7.8	0.007
**4o** mIPSC (Fast)	F _(1, 54)_ = 5.1	0.029	F _(1, 54)_ = 63.1	<0.0001	F _(1, 54)_ = 6.7	0.012
**4o** mIPSC (Slow)	F _(1, 54)_ = 12.3	0.001	F _(1, 54)_ = 29.6	<0.0001	F _(1, 54)_ = 7.4	0.0086
**6d**	F_(1,34)_ < 1	0.485	F_(1,34)_ = 23.8	<0.001	F_(1,34)_ = 13.4	<0.001
**6e**	F_(1,34)_ = 1.3	0.257	F_(1,34)_ = 15.4	<0.001	F_(1,34)_ = 7.5	0.010
**6f**	F_(1,34)_ = 3.5	0.069	F_(1,34)_ = 4.2	0.048	F_(1,34)_ < 1	0.391

To clarify whether Nlgn2 and/or MDGA1 selectively regulate the composition of postsynaptic sites, or whether they also play a role at presynaptic terminals, we performed immunohistochemical analysis for the presynaptic vesicular inhibitory amino acid transporter (VIAAT). Consistent with previous reports^9,11^, deletion of Nlgn2 did not alter size or number of VIAAT puncta (Fig. 2c–h, grey bars, and Supplementary Table 2). In contrast, deletion of MDGA1 resulted in a small but significant decrease in the size of VIAAT puncta, most prominently in layers S.R., and S.O., to a lesser extent in layer S.L.M., but not in layer S.P. (Fig. 2c–h, dark blue bars, and Supplementary Table 2). This pattern is highly consistent with the differential expression of MDGA1 in these hippocampal layers. Together, our findings indicate that Nlgn2 and MDGA1 mediate largely distinct and layer-specific effects at GABAergic synapses in hippocampal area CA1, which reflect their differential expression in the respective layers. Interactions between Nlgn2 and MDGA1 function at postsynaptic sites are limited to layer S.R., where the highest degree of colocalization of Nlgn2 with MDGA1 is observed.

### MDGA2 and Nlgn2 functionally interact to regulate GABA_A_R abundance in area CA1

While most evidence indicates that MDGA1, but not MDGA2, plays an important role at hippocampal GABAergic synapses^25,26^, MDGA2 was also reported to be present at GABAergic synapses in dissociated neuron cultures^32^. Unfortunately, the lack of a suitable antibody prevented us from assessing Nlgn2-MDGA2 colocalization in situ. To nevertheless determine whether MDGA2 modulates Nlgn2 functions at GABAergic synapses in hippocampal area CA1, we immunohistochemically assessed gephyrin, GABA_A_Rγ2 and VIAAT in Nlgn2 KO/MDGA2 heterozygous KO mice (since homozygous MDGA2 KO is lethal^26^). Intriguingly, MDGA2 Het mice displayed a reduction of the number and size of GABA_A_Rγ2 puncta that was most prominent in the S.P., and that was exacerbated in Nlgn2 KO/MDGA2 Het mice (Supplementary Table 3). No relevant effects on gephyrin or VIAAT were observed. Although it is difficult to interpret the reduction in GABA_A_Rγ2 puncta without knowing in which layers MDGA2 is expressed, it is conceivable that MDGA2 functionally replaces MDGA1 in layer S.P., from which MDGA1 is mostly absent.

### MDGA1 regulates the formation of Nlgn2 KO-related extrasynaptically-located gephyrin aggregates

Beyond the effects of Nlgn2 KO on GABAergic synapses, a striking and robust observation in Nlgn2 KO mice is the presence of prominent cytoplasmic gephyrin aggregates that are found at the border between layers S.P. and S.O.^11^. These aggregates were proposed to result from a loss of nucleation sites for GABAergic postsynapses in the absence of Nlgn2, leading to disrupted gephyrin transport to synaptic sites^11^, but whether other molecules are involved in the regulation of cytoplasmic gephyrin aggregates remains to be determined.

**Fig. 3:**
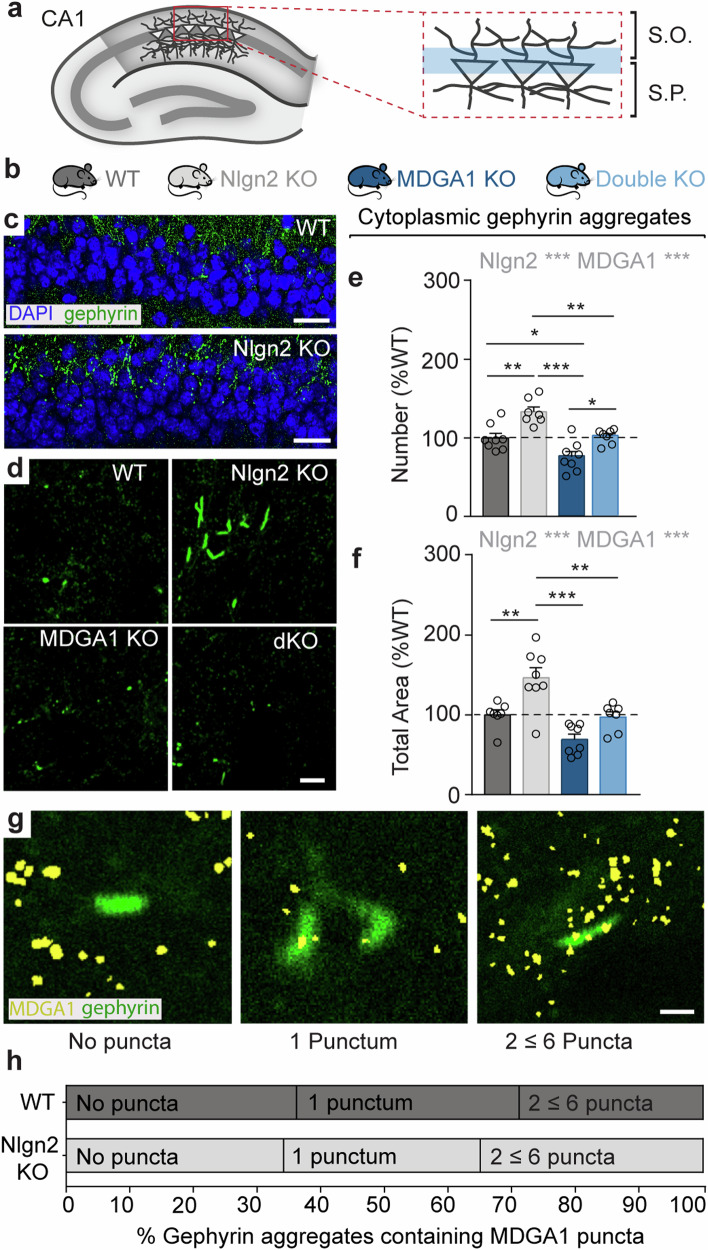
MDGA1 regulates the formation of Nlgn2 KO-related cytoplasmic gephyrin aggregates. **a** Schematic diagram of the dorsal hippocampus showing the region at the border between S.P. and S.O. in which gephyrin aggregates were detected. **b** Schematic representation of the four genotypes analyzed in this study. **c** Photomicrographs of the border region between S.P. and S.O. labelled with DAPI (blue) and anti-gephyrin antibody (green) in WT and Nlgn2 KO mice. Scale bars 25 µm. **d** High magnification photomicrographs of gephyrin aggregates in WT, Nlgn2 KO, MDGA1 KO and Nlgn2/MDGA1 dKO mice. Scale bar 5 µm. **e**, **f** Quantification of the number and total area of gephyrin aggregates, expressed as percentage of WT. **g** High magnification photomicrographs of gephyrin aggregates (green) showing the degree of co-localization with MDGA1 puncta (yellow) when no puncta, 1 punctum and 2 ≤ 6 puncta were detected and counted. **h** Schematic representation of the degree of co-localization of MDGA1 puncta in gephyrin aggregates in WT and Nlgn2 KO mice expressed as percentage of puncta detected. Statistically significant ANOVA comparisons are marked in gray at the top of panels and listed in Table 1. For all other ANOVA comparisons, F < 1. Post-hoc analysis (Tukey’s multiple comparison test): *p < 0.05, **p < 0.01, ***p < 0.001. Error bars represent SEM, and each circle represents an experimental animal (*n* = 7–8).

To investigate an involvement of MDGAs in this process, we tested whether MDGA1 or MDGA2 alone engage in the formation of these gephyrin aggregates, and whether they influence aggregate formation in Nlgn2 KO mice (Fig. 3a, b). As expected based on previous reports^11^, a robust increase in the number and in the total area of extrasynaptic cytoplasmic gephyrin aggregates was observed in putative CA1 pyramidal cell dendrites at the border between layers S.P. and S.O. in Nlgn2 KO mice (Fig. 3c–f, grey bars, and Table 1). Strikingly, this increase was completely absent in Nlgn2/MDGA1 dKO mice, which showed exactly the same number and total area of aggregates as WT mice (Fig. 3c–f, light blue bars, and Table 1), indicating that deletion of MDGA1 reverses the effect of Nlgn2 deletion on gephyrin aggregation. In contrast, MDGA2 heterozygous deletion had no effect on gephyrin aggregation, and Nlgn2 KO/MDGA2 Het mice showed the same number and total area of aggregates as Nlgn2 KO mice (Supplementary Fig. 3a–c, and Supplementary Table 7).

To establish whether MDGA1 is localized to gephyrin aggregates, where it may actively modulate their formation and extrasynaptic localization, we quantified the degree of co-localization between MDGA1 and gephyrin aggregates (Fig. 3g, h). In both WT and Nlgn2 KO mice, approximately 35% of gephyrin aggregates contained no MDGA1 puncta, 30–35% contained one MDGA1 punctum, and the remainder contained two or more MDGA1 puncta (Fig. 3h). No difference was observed between WT and Nlgn2 KO mice. These data indicate that while MDGA1 may be present in some gephyrin aggregates, its presence is not required to retain gephyrin in these aggregates, and that it must therefore act through another as yet unknown mechanism.

### Loss of MDGA1 expression perturbs GABAergic synaptic transmission in CA1 pyramidal neurons

**Fig. 4:**
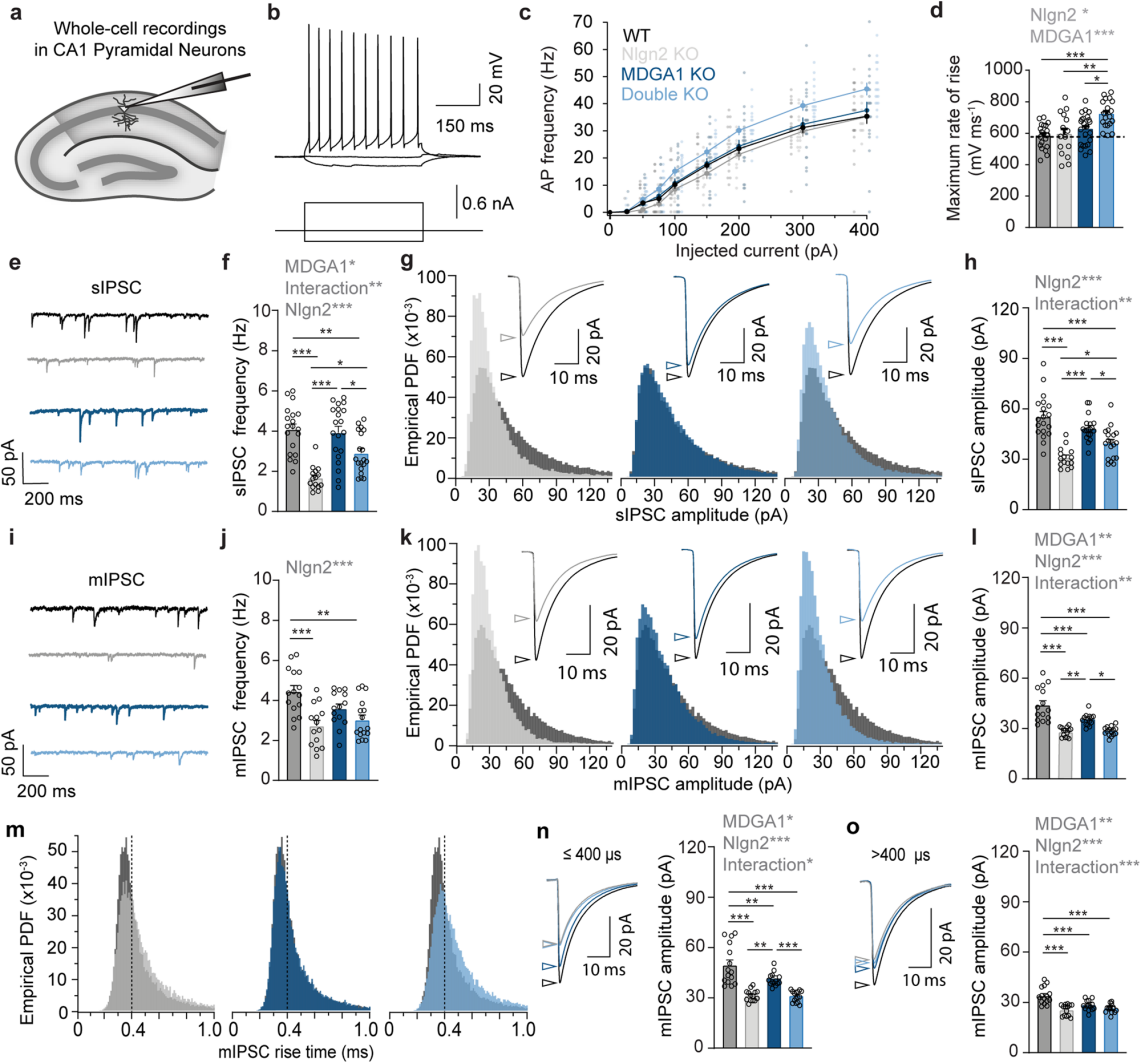
Loss of MDGA1 expression perturbs spontaneous GABAergic transmission in hippocampal CA1 pyramidal neurons. **a** Schematic diagram of the experimental paradigm for recording sIPSCs and mIPSCs in CA1 pyramidal neurons of the adult dorsal hippocampus. **b** Representative firing pattern of a CA1 pyramidal neuron. **c** Mean frequency of action potentials (APs) in response to steps of injected current. **d** Bar graphs and scatter plots showing means and individual values, respectively, for the maximal rate of AP rise in CA1 pyramidal neurons of WT, Nlgn2 KO, MDGA1 KO, Nlgn2/MDGA1 dKO mice. **e** Representative sIPSCs recorded in the four genotypes, color-coded as in **c**. **f** Bar graphs and scatter plots showing means and individual values, respectively, for sIPSC frequency. **g** Empirical probability density functions (PDFs) of individual sIPSC amplitudes in CA1 pyramidal neurons of mutant (light gray or blue) in comparison to WT (dark gray) mice. **h** Bar graphs and scatter plots showing grand averages and mean values for individual CA1 neurons, respectively, for sIPSC amplitudes. **i** Representative mIPSCs recorded in the four genotypes color-coded as in **c**. **j** Bar graphs and scatter plots showing means and individual values, respectively, for mIPSC frequency. **k** Empirical PDFs of individual mIPSC amplitudes in CA1 pyramidal neurons of mutant (light gray or blue) in comparison to WT (dark gray) mice. **l** Bar graphs and scatter plots showing grand averages and mean values for individual CA1 neurons, respectively, for mIPSC amplitudes. **m** Empirical PDFs of individual mIPSC rise times in CA1 pyramidal neurons of mutant (light gray or blue) in comparison to WT (dark gray) mice. **n** Average waveforms (left) and mean amplitudes of mIPSCs (right) with rise times ≤400 µs, arising at putative proximal inputs including perisomatic GABAergic contacts. **o** Average waveforms (left) and mean amplitudes of mIPSCs (right) with rise times >400 µs, arising at putative distal dendritic inputs. Error bars represent SEM. Each circle in **d**, **f**, **h**, **j**, **l**, **n** and **o** represents data from one CA1 pyramidal neuron. Statistically significant ANOVA comparisons are marked in gray at the top of panels and listed in Table 1. For all other ANOVA comparisons, F < 1. Post-hoc analysis (Tukey’s multiple comparison test): *p < 0.05, **p < 0.01, ***p < 0.001. In bar graphs, each circle represents a single cell (*n* = 14-24 cells for APs and rate of rise; 14-20 cells for sIPSC recordings; 14-16 cells for mIPSC recordings; four animals per genotype).

To determine whether the observed alterations in gephyrin and GABA_A_Rγ2 puncta affect inhibitory synaptic transmission in hippocampal area CA1, we performed whole-cell patch-clamp recordings in pyramidal neurons of acute hippocampal slices obtained from adult (6-8 week old) mice (Fig. 4a).

CA1 pyramidal neurons were identified based on their location, morphology and firing pattern (Fig. 4b). Their resting membrane potential and passive membrane properties were similar in all genotypes (Supplementary Table 4). While action potential (AP) firing threshold, AP amplitude, and AP half width were unchanged (Supplementary Table 4), CA1 pyramidal cells in Nlgn2/MDGA1 dKO mice showed a surprising tendency towards higher AP frequency in response to injection of depolarizing current steps as compared to other genotypes (Fig. 4c, light blue trace). Analysis of AP kinetics revealed a slightly increased maximal rate of rise in CA1 pyramidal cells of Nlgn2/MDGA1 dKO mice (Fig. 4d, light blue bars, and Table 1). No differences in passive membrane properties and discharge behavior were detected in CA1 pyramidal cells in MDGA2 Het mice (Supplementary Fig. 3d-f, and Supplementary Table 4).

In line with previous observations^11^, the frequency and amplitude of spontaneously occurring inhibitory postsynaptic currents (sIPSCs; recorded in the absence of TTX) and miniature inhibitory postsynaptic currents (mIPSCs; recorded in the presence of TTX) were strongly reduced in CA1 pyramidal cells of Nlgn2 KO mice (Fig. 4e–l, grey traces, Supplementary Fig. 4a–d and Table 1). Surprisingly, we also observed a significant reduction of mIPSC amplitude in CA1 pyramidal cells of MDGA1 KO mice (Fig. 4l, Supplementary Fig. 4c, Supplementary Fig. 4b, and Table 1), in contrast to previous studies reporting augmentation of GABAergic inhibition following MDGA1 KO^25^. Interestingly, sIPSC frequency and amplitude partially rescued to near WT-like levels in CA1 pyramidal cells of Nlgn2/MDGA1 dKO mice (Fig. 4e–h, Supplementary Fig. 4a, b, light blue traces and bars, and Table 1). In contrast, loss of a single MDGA2 allele had no major effects on GABAergic transmission (Supplementary Fig. 3f–k, dark green bars, and Supplementary Table 7), nor did it rescue the functional deficits observed in Nlgn2 KO mice (Supplementary Fig. 3f–k, light green, and Supplementary Table 7). Together, these findings indicate that loss of MDGA1 expression, but not heterozygous expression of MDGA2, slightly reduces GABAergic synaptic transmission, while combined loss of MDGA1 and Nlgn2 partially reverses the profound defects of GABAergic synaptic transmission observed after Nlgn2 KO.

Given the preferential localization of MDGA1 to layer S.R. compared to layer S.P. and the fact that MDGA1 deletion affected GABAergic synapse markers most prominently in layer S.R., it is conceivable that synaptic transmission may also be differentially affected in a layer-specific manner. To determine whether mIPSCs arising at different synaptic locations are differentially affected by MDGA1 loss, we took advantage of the fact that mIPSCs arising at proximal synapses, including perisomatic GABAergic contacts in layer S.P., are expected to have faster kinetics compared to those arising at distal dendritic synapses, such as those in layer S.R^47–49^. We, therefore, selectively analyzed mIPSCs separated by their rise times (Fig. 4m–o). Comparison of rise-time histograms obtained from recordings in mutant vs WT mice (Fig. 4m) revealed a shift towards slower rising mIPSCs in Nlgn2 KO and Nlgn2/MDGA1 dKO mice, consistent with a preferential loss of fast rising mIPSCs (≤400 µs rise time). Such a shift in the distribution of mIPSC rise times was not observed in MDGA1 KO mice. We then quantified changes in mIPSC amplitude for fast rising (≤400 µs rise time; Fig. 4n) and slowly rising (>400 µs rise time; Fig. 4o) mIPSCs. Consistent with the Nlgn2 loss-induced changes to mIPSC rise-time histograms, Nlgn2 KO mice showed a more pronounced amplitude reduction of fast rising mIPSCs compared to those with slow rise times, supporting the notion of a preferential effect of Nlgn2 on perisomatic synapses. However, amplitudes of fast and slowly rising mIPSCs were similarly affected in MDGA1 KO mice, indicating that MDGA1 does not result in layer-specific alterations in synaptic transmission when assayed by separating mIPSCs by their rise times.

### Loss of MDGA1 expression does not affect excitatory synapses in area CA1

**Fig. 5:**
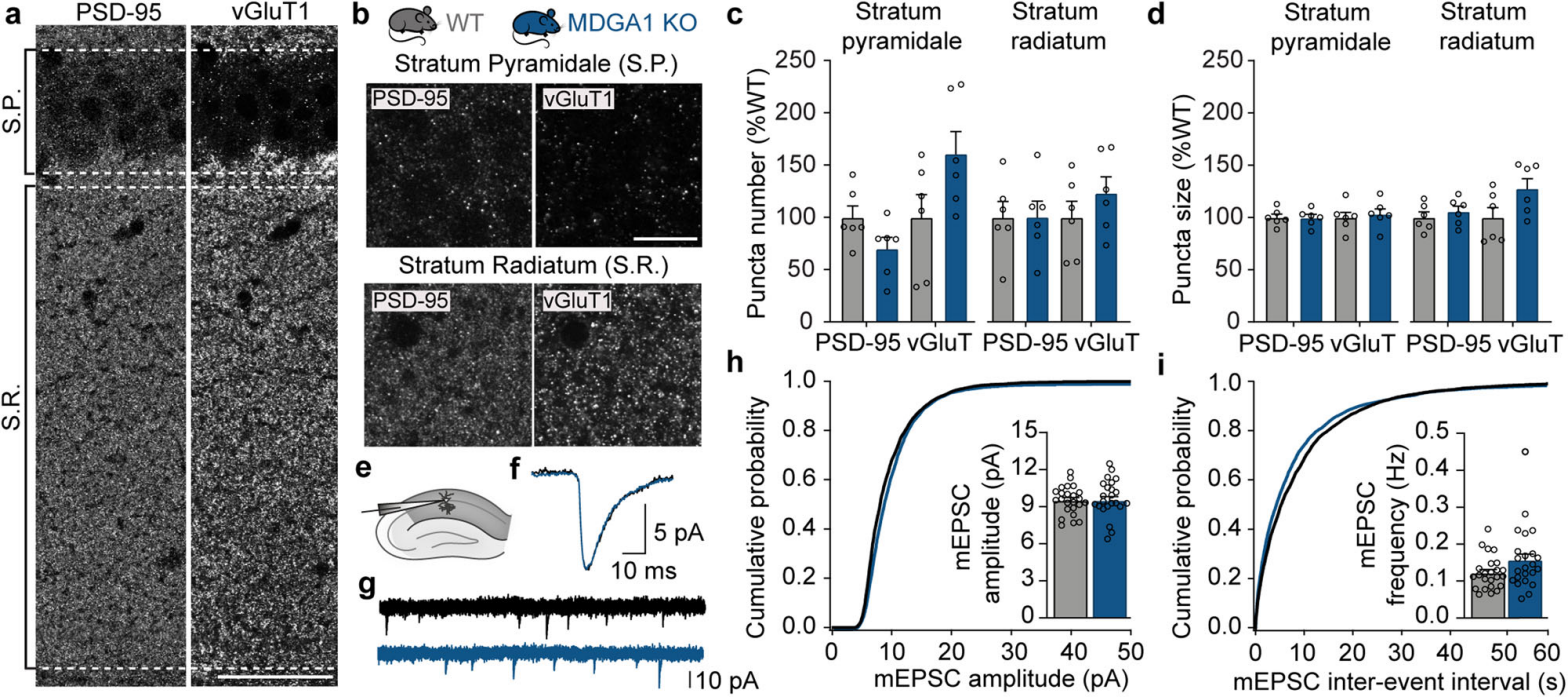
Loss of MDGA1 expression does not affect excitatory neurotransmission in area CA1. **a** Photomicrographs showing an overview of layers Stratum pyramidale (S.P.) and Stratum radiatum (S.R.) in a hippocampal section of a WT mouse labelled with antibodies against PSD-95 (left) and vGluT1 (right). Scale bar 50 µm. **b** Schematic representation of the two genotypes tested (WT and MDGA1 KO) and high magnification photomicrographs of layer S.P. and S.R. stained for PSD-95 and vGluT1. Scale bar 5 µm. **c** Bar graphs and scatter plots showing means and individual values, respectively, for the number of PSD-95 and vGluT1 puncta in S.P. and S.R. **d** Bar graphs and scatter plots showing means and individual values, respectively, for the size of PSD-95 and vGluT1 puncta in S.P. and S.R. **e** Schematic diagram of the experimental paradigm for recording mEPSCs in CA1 pyramidal neurons of the adult dorsal hippocampus. **f** Representative average waveforms of mEPSCs recorded in CA1 pyramidal neurons of WT (black traces) and MDGA1 KO (blue traces) mice. **g** Four consecutive mEPSC recording sweeps shown superimposed for WT (black) and MDGA1 KO (blue) recordings. Same cells as shown in **f**. **h** Average cumulative distributions of individual mEPSC amplitudes. Inset: Bar graphs and scatter plots showing grand averages and individual values, respectively, for mEPSC mean amplitudes. **i** Average cumulative distributions of individual mEPSC inter-event intervals. Inset: Bar graphs and scatter plots showing grand averages and individual values, respectively, for mEPSC mean frequencies. Statistical differences were assessed with unpaired *t*-tests Error bars represent SEM, each circle represents an experimental animal in **c**, **d**, and a single-cell in **h**, **i**. Differences were not significant in any comparison.

Given the surprising controversies among previous reports about whether MDGA1 primarily modulates the function of inhibitory^25,31^ or excitatory^33,50^ synapses, we next tested the effect of MDGA1 loss on excitatory synapses in the CA1 region of the adult hippocampus. Immunohistochemical analysis for the excitatory synapse-specific scaffolding protein PSD-95 and the presynaptic vesicular glutamate transporter vGluT1 revealed no significant differences between WT and MDGA1 KO mice with respect to the number or size of PSD-95 and vGluT1 puncta in layers S.R., S.P., and S.L.M. (Fig. 5a–d and supplementary Table 5). Interestingly, a small increase in the size of vGluT1 puncta, but not PSD-95 puncta, was observed in layer S.O., indicating either minor effects on a small subset of excitatory synapses or compensatory changes (Supplementary Table 5). Functional analysis of excitatory synaptic transmission revealed no significant changes in the amplitude or frequency of spontaneously occurring miniature excitatory postsynaptic currents (mEPSCs) (Fig. 5e–i). Taken together, our data demonstrate that loss of MDGA1 expression has virtually no effect on spontaneous excitatory synaptic transmission, but selectively affects inhibitory GABAergic synapses in the CA1 subfield of the adult hippocampus.

### MDGA1 deletion ameliorates abnormal anxiety-related avoidance behavior in female Nlgn2 KO mice

**Fig. 6:**
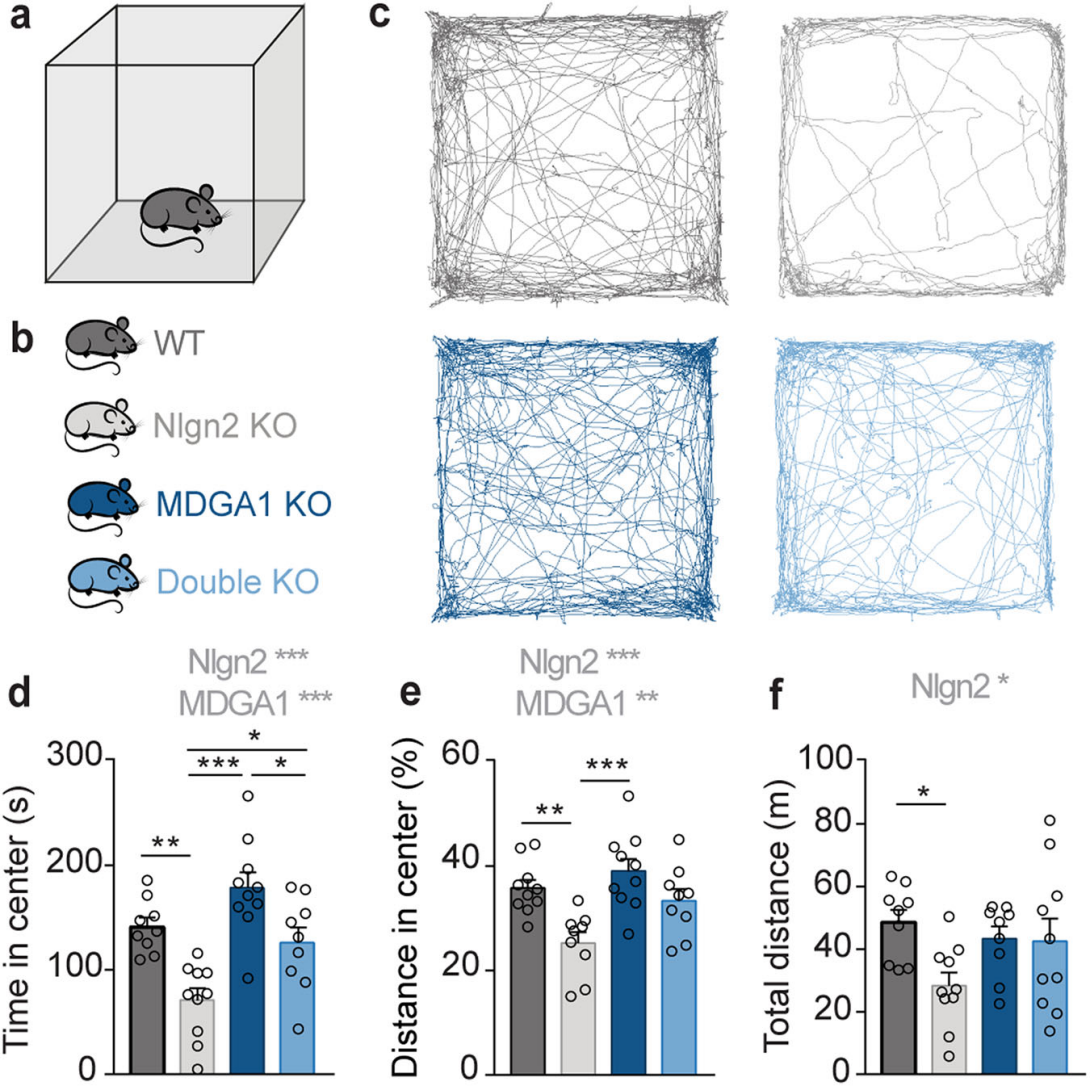
MDGA1 deletion ameliorates abnormal anxiety-related avoidance behavior in female Nlgn2 KO mice. Schematic representation of the OF arena (**a**) and of the four experimental genotypes analyzed (**b**). **c** Representative tracks of OF exploration. **d** Time spent in the anxiogenic region (center) of the OF arena. **e** Distance traveled in the center of the OF, expressed as percentage of total distance traveled. **f** Total distance travelled in the OF. Statistically significant ANOVA comparisons are marked in gray at the top of panels and listed in Table 1. For all other ANOVA comparisons, F < 1. Post-hoc analysis (Tukey’s multiple comparison test): *p < 0.05, **p < 0.01, ***p < 0.001. Error bars represent SEM, and each circle represents an experimental animal (*n* = 9–10).

Finally, in light of the role of both Nlgn2 and MDGA1/2 in the pathophysiology of psychiatric disorders^34–43^, we assessed how the functional interaction between Nlgn2 and MDGAs might influence psychiatrically relevant behaviors. Nlgn2 KO in mice was shown to cause a profound increase in anxiety-related avoidance behaviors in several tests, including the open field test, the elevated plus maze, and the light/dark box^9,46,51^, which at least partially originates from altered connectivity in hippocampal-amygdala-prefrontal circuits^45^. In the present study, we used an open field task to test whether this anxiety phenotype is modulated in Nlgn2/MDGA1 dKO or Nlgn2 KO/MDGA2 Het mice (Fig. 6a–c, Supplementary Fig. 5a-c). Strikingly, but consistent with the amelioration of the defects in gephyrin aggregation and sIPSC frequency and amplitude, female, Nlgn2/MDGA1 dKO mice showed an amelioration of the profound Nlgn2 KO anxiety phenotype as indicated by a normalization of the time spent in the center of the open field chamber (Fig. 6d, light blue bars vs. grey bars, and Supplementary Table 7). Surprisingly, however, male Nlgn2 KO mice in this experiment did not display the anxiety phenotype previously observed, likely due to complex interactions with strain background or parental behavior of the Nlgn2 Het/MDGA1 Het breeders (Supplementary Fig. 5d, e and Supplementary Table 7). Accordingly, we were unable to determine whether the anxiety phenotype of Nlgn2 KO mice is also ameliorated in male Nlgn2/MDGA1 dKO mice. No amelioration of the Nlgn2 KO anxiety phenotype was observed in male or female Nlgn2 KO/MDGA2 Het mice (Supplementary Fig. 5f-h and Supplementary Table 7), consistent with the lack of an effect of MDGA2 Het on gephyrin aggregation and GABAergic synaptic transmission (Supplementary Fig. 5a–c and Supplementary Table 7).

Taken together, our observations indicate functional interactions between MDGA1 and Nlgn2 KOs, but not between MDGA2 and Nlgn2 KOs, in modulating the neuronal circuits that regulate anxiety-related avoidance behaviors. Further, our data reveal an anxiolytic effect of MDGA1 deletion in Nlgn2 KO mice, raising the intriguing possibility that the MDGA1-Nlgn2 functional interaction may serve as a novel target for anxiolytic therapeutic strategies.

## Discussion

In the present study, we sought to determine how the schizophrenia- and autism-associated synaptic adhesion proteins Nlgn2 and MDGAs functionally interact in vivo to regulate GABAergic synapses. We report that Nlgn2 and MDGA1 show distinct distribution patterns in hippocampal area CA1, with Nlgn2 localized in a punctate pattern that was most prominent in layers S.P. and S.L.M., and MDGA1 distributed diffusely throughout this region, most strongly in layers S.R. and S.O. Using immunolabeling, electrophysiological and behavioral analysis in Nlgn2/MDGA1 dKO mice, we found that the combined loss of Nlgn2 and MDGA1 leads to an exacerbated reduction of gephyrin puncta specifically in layer S.R. At the same time, a normalization of cytoplasmic gephyrin aggregates at the S.P.-S.O. boundary was observed in Nlgn2/MDGA1 dKO mice, accompanied by a partial normalization of defects in sIPSC characteristics in CA1 pyramidal neurons and of the anxiety-related behavior in an open field test. Importantly, loss of MDGA1 selectively affected inhibitory but not excitatory synapses, with virtually no differences observed in PSD-95 and vGluT1 puncta or in the amplitude or frequency of mEPSCs. Heterozygous KO of MDGA2 in Nlgn2 KO mice had only very subtle effects on GABAergic synapses, indicating that either a single MDGA2 allele suffices or that MDGA2 plays a minor role in modulating Nlgn2 function. Together, our experimental data indicate that the key function of the interaction between Nlgn2 and MDGA1 bidirectionally regulates gephyrin aggregation in the intact hippocampal area CA1 network, which in turn determines the effects of these proteins on GABAergic synapse assembly and function and on anxiety-related behavior.

While extensive structural data document a molecular interaction between Nlgn2 and MDGAs in vitro, it has to date remained unknown to which extent and where these proteins colocalize in situ. Here we report that in hippocampal area CA1 of adult mice, MDGA1 shows a relatively diffuse staining pattern, which is most prominent in layers S.R. and S.O., and to a lesser extent in layer S.L.M., while it is largely absent from layer S.P. In contrast, Nlgn2 is present in a punctate pattern, consistent with previous reports^11^, with a particularly prominent staining in layers S.P. and S.L.M. and a lesser, albeit still strong, staining in layers S.O. and S.R. Accordingly, the strongest colocalization of Nlgn2 with MDGA1 is observed in layer S.R., as well as in layers S.O. and S.L.M., while very little colocalization was observed in layer S.P. Consistent with their expression, the most pronounced effects of MDGA1 KO and Nlgn2/MDGA1 dKO were observed in the S.R., with a significant reduction of GABA_A_Rγ2 in both MDGA1 KO and Nlgn2/MDGA1 dKO mice, and an exacerbation of gephyrin loss in the Nlgn2/MDGA1 dKO. Whether this potentiation of the loss of gephyrin clusters in Nlgn2/MDGA1 dKO mice results from a direct molecular interaction of the two proteins, or whether it reflects independent regulatory pathways, cannot easily be distinguished. Nevertheless, our findings identify layer S.R. as one of the primary sites of the combined function of Nlgn2 and MDGA1 at GABAergic synapses in area CA1. Layer S.R. consists primarily of dendritic arborizations of pyramidal neurons with a cell body in layer S.P., and this layer is the primary area of excitatory input from area CA3 through the Schaffer collateral pathway, placing the Nlgn2-MDGA1 functional interaction in an ideal position to modulate the flow of information through this pathway. A number of different GABAergic neuron subtypes target the dendritic tree of CA1 pyramidal neurons specifically in layer S.R., including parvalbumin-positive bistratified cells, neuropeptide Y-positive Ivy cells, and cholecystokinin-positive Schaffer collateral-associated and apical dendrite-innervating cells^44^. Whether Nlgn2 and/or MDGA1 loss selectively or predominantly affects any of these inputs remains to be determined.

A second striking effect of the Nlgn2/MDGA1 dKO is the normalization of the number of the cytoplasmic gephyrin aggregates that occur upon Nlgn2 KO at the boundary between layers S.P. and S.O^11^. This extrasynaptic cytoplasmic gephyrin aggregation is thought to result from the loss of the gephyrin-collybistin-Nlgn2 triad, which is necessary for Nlgn2-mediated recruitment of gephyrin to GABAergic synapses and the consequent somatic gephyrin accumulation^11^. However, it is unknown whether these aggregates consist exclusively of gephyrin, and why they are localized so specifically at the boundary between layers S.P. and S.O. rather than being distributed throughout the cytoplasm of CA1 pyramidal cells. Moreover, our data from Nlgn2/MDGA1 dKO mice indicate that the presence of these cytoplasmic gephyrin aggregates appears to be unrelated to the localization of gephyrin and GABA_A_Rγ2 at synapses, since the number of gephyrin aggregates is normalized in these mice (Fig. 3c–e), while the loss of synaptic gephyrin and GABA_A_Rγ2 clusters in S.R. is exacerbated (Fig. 2f–h). The normalization of the cytoplasmic gephyrin aggregates correlates well with the partial normalization of sIPSC frequency and amplitude, as well as with the partial normalization of the anxiety behavior in the female Nlgn2/MDGA1 dKO mice. These data lead to the fascinating conclusion that it is this reduction of gephyrin aggregates, rather than the exacerbation of the loss of gephyrin from layer S.R., that dominates the functional consequences of combined deletion of Nlgn2 and MDGA1. The mechanistic link between the cytoplasmic gephyrin aggregates and the functional consequences at the cellular and behavioral level, as well as the reason for the apparent discrepancy between gephyrin aggregate formation and loss of synaptic gephyrin, remains to be established. Thus, it will be interesting to determine whether the pathophysiological consequences of gephyrin aggregation may stem from a gain-of-function mechanism of the aggregates themselves, or whether normalization of the aggregates may result in the correct integration of GABAergic synapse components other than the ones assessed in our immunohistochemical analysis. Regional differences in the expression of gephyrin splice forms^52^ or the differential recruitment and trafficking of GABA_A_R subunits other than GABA_A_Rγ2 may also play a role.

Our findings additionally raise the intriguing question as to how MDGA1 differentially regulates the number of synaptic gephyrin and GABA_A_Rγ2 clusters vs. the cytoplasmic gephyrin aggregates. Co-staining of Nlgn2 and MDGA1 indicates that MDGA1 is not strictly localized to Nlgn2-positive clusters, but rather displays a relatively diffuse distribution that partially overlaps with Nlgn2. This is consistent with recent findings in neuronal cultures indicating that MDGAs are homogeneously distributed over the cell surface and exhibit fast diffusion throughout the dendritic membrane, where they interact with Nlgn1 extrasynaptically and prevent Nlgn1 and AMPARs from entering nascent glutamatergic synapses^33^. Based on our immunolabeling analysis, it is plausible that a similar mechanism holds true for the Nlgn2-MDGA1 interaction, and that direct molecular interactions primarily take place extrasynaptically to regulate the trafficking of GABAergic synapse components to synaptic sites. At the same time, it was recently shown that Nlgn2 is not the only target of MDGA1, and that a transsynaptic interaction between MDGA1 and APP regulates synapse function independently of Nlgn2 at GABAergic synapses formed by oriens-lacunosum moleculare (O-LM) interneurons onto the distal dendrites of pyramidal neurons in layer S.L.M^31^. It is conceivable that the additive effects of Nlgn2/MDGA1 dKO in layer S.R. results from the loss of an MDGA1 interaction with an unidentified additional synaptic partner, independently of Nlgn2. In contrast, the normalization of gephyrin aggregation in the Nlgn2/MDGA1 dKO mice likely results from the loss of a direct antagonistic interaction between these two proteins. Our observation that not all cytosolic gephyrin aggregates contain MDGA1, and that loss of Nlgn2 does not affect the degree of co-localization between MDGA1 and gephyrin aggregates, indicates that MDGA1 is not directly involved in the retention of gephyrin in extrasynaptic aggregates. Rather, it may bind to and sequester other proteins that contribute to the membrane trafficking and synaptic localization of gephyrin. Since MDGA1 can also bind to Nlgn1^33^ and potentially other Nlgns, it is possible that additional KO of MDGA1 in the Nlgn2 KO mice releases an excess pool of another Nlgn isoform, which can then substitute for the absent Nlgn2 in disassembling gephyrin aggregates.

A further aim of this study was to establish whether MDGA1 and MDGA2 differ in their importance for the regulation of inhibitory transmission, since the relative effect of the two MDGAs on GABAergic synapse function has been controversially discussed. One set of studies on the hippocampal area CA1 indicated that deletion of MDGA1 causes an increase in mIPSC frequency but not in mEPSC frequency, and an increase in the number of symmetric but not asymmetric synapses identified in electronmicrographs^25^, while heterozygous deletion of MDGA2 causes morphological and functional alterations at glutamatergic but not GABAergic synapses^26,53^. These data led to the conclusion that MDGA1 and MDGA2 must be specific to inhibitory and excitatory synapses, respectively. In contrast, a proteomics study on cortical neuron cultures detected MDGA1 and MDGA2 in the excitatory and inhibitory synaptic clefts, respectively^32^, resulting in the opposite conclusion. Recent data from dissociated hippocampal cultures and organotypic slices cultures indicated no change in mIPSC frequency or amplitude following deletion of either MDGA1 or MDGA2^33^, leading the authors to conclude that during early development, neither MDGA protein plays a role at inhibitory synapses. In contrast, another recent report using dissociated hippocampal cultures from conditional MDGA knockout mice supported the original notion that MDGA1 and MDGA2 specifically suppress the formation of inhibitory and excitatory synapses, respectively^27^. Moreover, the same group found that overexpression of WT MDGA1 and Nlgn2 binding-deficient MDGA1, but not APP binding-deficient MDGA1, causes a reduction in mIPSC frequency in hippocampal area CA1, while conditional deletion of MDGA1 in area CA1 had no effect on mIPSC frequency^31^. Most recently, a report using epitope-tagged knock-in mice proposes that MDGA2, but not MDGA1, regulates GABAergic inhibitory synapses, while both MDGAs regulate glutamatergic excitatory synapses^50^. These findings indicate that the effects of the MDGAs appear to depend on the experimental conditions, potentially due to differences in the ratio of MDGAs to Nlgns and other binding partners as shown previously^33^. These inconsistencies indicate an urgent need for further investigation of the synapse specificity of MDGA1 and MDGA2. Here we report subtle and layer-specific effects on GABA_A_Rγ2 localization in both MDGA1 KO and MDGA2 Het mice, but the most prominent effects on gephyrin aggregates, sIPSC and mIPSC frequency, were found almost exclusively in Nlgn2/MDGA1 dKO but not Nlgn2 KO/MDGA2 Het mice. Surprisingly, deletion of only MDGA1 resulted in a modest reduction in mIPSC amplitude as well as in a decreased size of VIAAT and GABA_A_Rγ2 puncta size, in stark contrast to the increase in mIPSC frequency and symmetric synapse density previously reported in area CA1^25^. The reason for this discrepancy most likely lies in differences in the strain background (C57BL/6JRj in our study, mixed C57BL/6NxJ in Connor et al.^25^.), which has previously been shown to modulate both MDGA2 function^54^ and GABA_A_Rα2 expression^55^. Importantly, our findings of a reduction of GABAergic synapse markers are consistent with the smaller mIPSC amplitudes measured in this study, and our observation of a prominent reduction of sIPSC and mIPSC frequency and amplitude, as well as gephyrin and GABA_A_Rγ2 staining intensity in the Nlgn2 KO, are consistent with previous findings^11^, validating our technical approach. Moreover, consistent with Connor et al.^5^., we observed no effects on the number and function of glutamatergic excitatory synapses. Together, our data indicate that in the hippocampal area CA1 of adult mice, (i) only MDGA1, but not MDGA2, functionally interacts with Nlgn2, (ii) MDGA1 deletion results in a weakening, rather than a strengthening, of GABAergic synapses, and (iii) MDGA1 deletion alone does not affect excitatory glutamatergic neurotransmission in area CA1.

The ultimate objective of our study was the identification of key mechanisms by which Nlgn2 and MDGAs contribute to relevant phenotypes of psychiatric disorders they have been linked to, such as anxiety, autism and schizophrenia^7,21,34–43^, and to determine how such mechanisms can be targeted to ameliorate pathophysiological behaviors. We show that combined deletion of Nlgn2 and MDGA1 results in a partial reversal of the prominent anxiety phenotype observed in female Nlgn2 KO mice, consistent with the normalization of gephyrin aggregates and the partial normalization of sIPSC frequency in CA1 pyramidal neurons. Given this correlation, it is conceivable that the formation of these gephyrin aggregates contributes to the anxiety phenotype in the Nlgn2 KO mice, and that reversing gephyrin aggregate formation may ameliorate this behavior. A further detailed understanding of how Nlgn2 and MDGA1 differentially regulate gephyrin aggregation, and how these aggregates affect GABAergic synapse function, may therefore be relevant towards identifying pathophysiological mechanisms in Nlgn2- and MDGA1-related psychiatric disorders.

## Methods

### Experimental subjects

Neuroligin-2 knockout (Nlgn2 KO) mice^56^ were generated in our laboratory at the Max Planck Institute for Multidisciplinary Sciences (formerly Max Planck Institute of Experimental Medicine) and were maintained on a C57BL/6JRj background (Janvier Labs). MDGA1 knockout^57^ and MDGA2 heterozygous knockout^26^ mice on a C57BL6 background were generously provided by Tohru Yamamoto, and they were imported to the Max Planck Institute for Multidisciplinary Sciences via the laboratory of Ann Marie Craig, University of British Columbia. The mouse lines were crossed to generate Nlgn2/MDGA1 Het or Nlgn2/MDGA2 Het mice, and they were then backcrossed an additional 5-6 generations to a C57BL6/JRj background. For experiments involving MDGA1, Nlgn2/MDGA1 double Het parents were crossed to generate experimental cohorts consisting of littermates of four genotypes, i.e. WT, Nlgn2 KO, MDGA1 KO and Nlgn2/MDGA1 dKO mice. For experiments involving MDGA2, the breeding strategy needed to be adjusted, since homozygous deletion of MDGA2 is lethal^26^. Therefore, one Nlgn2 Het/MDGA2 Het parent was crossed with one Nlgn2 Het/MDGA2 WT parent to generate experimental cohorts consisting of littermates of four genotypes, i.e. WT, Nlgn2 KO, MDGA2 Het and Nlgn2 KO/MDGA2 Het mice. Animals were group-housed (2–4 mice per cage) and maintained on a 12 h light/dark cycle, with food and water ad libitum, and all experiments were performed during the light cycle. Male and female mice were used for all experiments in strict sets of four sex-matched mice, one of each experimental genotype, that were strictly processed together as a set from initiation of data acquisition to completion of data analysis. An ANCOVA analysis revealed no effect of sex as a co-variate for immunohistochemistry and electrophysiology experiments, while significant effects of sex as a co-variate were observed in the behavioral experiments. Therefore, behavior data, but not immunohistochemistry and electrophysiology data, were analyzed separately for male and female mice. For immunohistochemistry and behavior experiments, mice were 8–12 weeks old at the beginning of the experiment, while for electrophysiology experiments, mice were 6–8 weeks old. Experimenters were blind to genotype during all stages of data acquisition and analysis. All procedures were approved by the state of Niedersachsen (Landesamt für Verbraucherschutz und Lebensmittelsicherheit, license number 33.19-42502-04-18/2957) and followed the guidelines of the welfare of experimental animal use issued by the federal government of Germany and the Max Planck Society. We have complied with all relevant ethical regulations for animal use.

### Immunohistochemistry

Adult mice (8–12 weeks old) were anesthetized with isoflurane, decapitated, and their brains were rapidly dissected and immersed in isopentane at −35 to −38 °C for approximately 30 s. Brains were stored in the cryostat (Leica CM3050S, Leica Biosystems, Germany) for 30 min at −20 °C, after which 14–18 µm thick coronal brain sections were cut and mounted on glass slides. Brain sections were arranged in experimental sets containing sex-matched mice of all four genotypes that were processed together throughout the experiment, and they were mounted such that each glass slide contained exactly one section from each of the four experimental genotypes. Sections were dried at RT for 30 min and then fixed using one of two protocols, either methanol fixation or paraformaldehyde (PFA) post-fixation, depending on the antibody used. For methanol fixation (used for anti-Nlgn2, anti-MDGA1, anti-gephyrin and anti-GABA_A_Rγ2 antibodies), sections were immersed in methanol pre-cooled to −20 °C for 5 min, followed by 3 × 10 min washes with PBS. For PFA post-fixation (used for the anti-VIAAT, PSD-95, and vGluT1 antibodies), sections were incubated in 4% PFA in 0.1 M Sorensen’s phosphate buffer (pH 7.5) for 10 min at RT, followed by 2 × 10 min washes with PBS and 1 × 10 min wash with sodium citrate buffer (10 mM sodium citrate, 0.05% Tween-20, pH 8.0). They were then subjected to an antigen retrieval procedure, in which they were incubated in sodium citrate buffer at 95 °C for 30 min, followed by a cooling period of 20 min and subsequently 2 × 10 min washes with PBS at RT. After either methanol fixation or PFA post-fixation, sections were incubated for 1 h in blocking buffer (3% bovine serum albumin, 10% goat serum and 0.3% Triton-X) at RT. Afterwards, sections were incubated overnight at 4 °C in primary antibody in blocking buffer. The following primary antibodies, all from Synaptic Systems (Göttingen, Germany), were used: guinea pig anti-Nlgn2 (1:1000, Cat# 129205); rabbit anti-MDGA1 (1:2000, Cat# 421002); mouse anti-gephyrin (1:2000, Cat. # 147111); rabbit anti-GABA_A_Rγ2 (1:2000, Cat# 224003); mouse anti-VIAAT (1:2000, Cat# 131011), rabbit anti-PSD-95 (1:500, Cat# 124003); mouse anti-vGluT1 (1:2000, Cat# 135511).). On the next day, sections were washed 3 × 10 min with PBS and then incubated for 2 h with the following secondary antibodies (all from ThermoFisher Scientific, USA) in blocking buffer at RT: goat anti-guinea pig, A555 (1:1200, Cat # A21435); goat anti-rabbit A488 (1:1200, Cat # A11008), goat anti-rabbit A555 (1:1200, Cat # A21429), goat anti-mouse A488 (1:1200, Cat # A11029),, goat anti-mouse A633 (1:600, Cat # A21052), goat anti-mouse A555 (1:600, Cat# A21422), goat anti-rabbit A647 (1:600. Cat# A32733). Sections were then washed 2 × 10 min with PBS, incubated 10 min with DAPI (0.1 μg/ml, in PBS), washed 2 × 10 min PBS, and stored overnight at 4 °C to dry. Finally, they were coverslipped using Aqua-Poly/Mount mounting medium (Polysciences, Inc, USA).

### Image acquisition and processing for analysis of Nlgn2 and MDGA1 colocalization

Image acquisition for analysis of Nlgn2 and MDGA1 colocalization was conducted using a Leica TCS-SP8 laser scanning confocal microscope (Leica microsystems, Germany) equipped with a white light laser (WLL) and hybrid detectors (HyD). A 63× oil immersion objective with a numerical aperture of 1.4 was used to obtain single plane micrographs at 1024 × 1024 spatial resolution and pixel spacing of xy = 45.09 nm. Laser power was optimized to ensure that the detected fluorescence intensity is within the dynamic range of detection. All imaging parameters were kept constant for images acquired from WT and Nlgn2/MDGA1 dKO mouse brain sections and also for all images acquired from different HPC layers. Images were then subjected to deconvolution using the Lightning function of the Leica LAS X software (global mode). Tiled overview images of the hippocampus were acquired using the 20× oil immersion objective of the Leica SP8 (numerical aperture 0.75) where the navigator function of the LAS-X software (Leica microsystem, Germany) was used to acquire and stitch the tiles. Tiled overview images of the CA1 were acquired using the 63× objective.

Composite two-channel images of Nlgn2 and MDGA1 labelling acquired from different hippocampus layers were split and further processed using FIJI software (National Institute of Health, USA). Images of the Nlgn2 channel were binarized and subjected to noise despeckle and watershed segmentation in FIJI to retain clearly defined Nlgn2 puncta. The threshold value used for binarization of Nlgn2 images was calculated as follows: Threshold = 20×average intensity of images acquired from the KO sample mounted on the same slide as the WT sample used in the analysis. Binary images were then subjected to segmentation using the ‘analyze particles’ algorithm of FIJI using a size filter of 0.03-1.5 µm^2^ and were added to the ROI manager where the average intensity for every Nlgn2 punctum was measured inside the MDGA1 channel by using the ‘Measure’ command while redirecting the measurement settings to the MDGA1 image. Frequency distribution histograms of MDGA1 intensity inside Nlgn2 puncta across all images acquired (8 images per layer) were generated. Total area imaged per layer was 17060.74 µm^2^. High magnification photomicrographs in Fig. 1 and Supplementary Fig. 1 were processed by being subjected to contrast enhancement and smoothing (1×). The same minimum and maximum brightness range for every channel was used for all images taken from WT and dKO samples and for all images across hippocampus layers to allow for comparison. In order to calculate the percentage of Nlgn2 puncta colocalized with MDGA1 for every layer, a threshold of MDGA1 average fluorescence intensity was applied, defined as 10× the average intensity measured in images acquired from KO samples. The number of Nlgn2 puncta containing mean MDGA1 intensity above threshold was considered as colocalized and was then calculated as a percentage of the total number of Nlgn2 puncta detected per hippocampal layer and plotted as a doughnut chart using GraphPad Prism (GraphPad software, La Jolla, CA, USA).

### Image acquisition and processing for quantification of gephyrin, GABA_A_Rγ2, VIAAT, PSD-95, and vGluT1 puncta

Images of synaptic markers were obtained using the Leica TCS- SP8 laser scanning confocal microscope (Leica microsystems, Germany) equipped with a white light laser (WLL) and hybrid detectors (HyD), 63× oil immersion objective and 4× digital zoom at spatial resolution of (1024 × 1024 pixels). Within each set of four mice, sections were anatomically matched and settings for laser power, gain and offset were kept constant during image acquisition. For each animal, 12 z-stacks, each containing 4-5 optical sections, were obtained from each layer of the dorsal hippocampus CA1 region.

For analysis of Gephyrin, GABA_A_Rγ2 and VIAAT puncta in hippocampal CA1 layers S.O., S.R. and S.L.M., images were binarized using a threshold value that was applied to all images obtained from the same experimental set of four mice. To determine the threshold value for each set, background intensity for every image was manually measured and averaged across all images belonging to the same set, and the threshold for the entire set was defined at 3x background intensity for gephyrin, GABA_A_Rγ2, and VIAAT puncta, 2× background intensity for PSD-95 and vGluT1, and 10× background intensity for gephyrin aggregates. Binarized images were then subjected to noise despeckle and watershed segmentation algorithms in FIJI to reduce noise and improve segmentation to puncta. Next, images were subjected to “Analyze Particles” segmentation algorithm using a size filter of 0.04–1 µm^2^ for gephyrin puncta, 0.8-infinity for gephyrin aggregates, 0.04–3.25 µm^2^ for GABA_A_ R γ2 puncta, and 0.03 to 1.5 µm^2^ for PSD-95 and vGluT1 puncta. Total number, size and total intensity of puncta for every image were measured and average values were calculated per each experimental group and plotted for each hippocampal layer.

To quantify perisomatic synapses in layer S.P., the perisomatic area was manually identified by tracing the perimeter of the cell body (defined as a circular area devoid of immunofluorescence signals). The perimeter was then expanded by 1.4 µm in each direction for quantification of puncta in the perisomatic region of the outlined cell body. Synaptic puncta were quantified in the selected area using the “analyze particles” algorithm in FIJI. Number and total area of particle were normalized by the perimeter length of the cell.

### Image acquisition and processing for analysis of colocalization of MDGA1 and gephyrin aggregates

Image acquisition for the analysis of MDGA1 and gephyrin aggregates was performed using a Leica SP8 Confocal microscope (Leica microsystems, Germany), equipped with Photomultiplier tube (PMT) and Hybrid (HyD) detectors. A 63X oil immersion objective with a numerical aperture of 1.4 was used to obtain single plane micrographs at 1024 × 1024 spatial resolution. Images were obtained from the interface of S.O. and S.P and the settings for laser power, gain and offset were kept constant for images acquired from WT and Nlgn2 KO mice.

To determine the threshold value for each set, background intensity for every image was manually measured and averaged across all images (WT and KO) belonging to the same set. The threshold for the entire set was defined at 2.5× background intensity for MDGA1 puncta and 2× background intensity for gephyrin aggregates. After setting the threshold, binarized images of the MDGA1 channel were subjected to noise despeckle and watershed segmentation in FIJI. Images were then subjected to the “Analyze Particles” segmentation algorithm using a filter size of 0.01–1.75 µm^2^ for MDGA1 puncta. The quantified MDGA1 puncta were superimposed with threshold-adjusted gephyrin aggregates, and colocalization of MDGA1 puncta and gephyrin aggregates was quantified manually.

### Quantitative real-time PCR

Hippocampi from WT, MDGA1 KO and MDGA2 Het mice were dissected, rapidly frozen in liquid nitrogen, and stored at −80 °C. Tissue homogenization was performed by cryomortar pulverization in liquid nitrogen. Total RNA was extracted from the hippocampal tissue powder using the RNeasy Mini Kit (Qiagen, Cat # 74104), following manufacturer’s instructions. Complementary DNA (cDNA) synthesis was carried out from 500 ng of total RNA using the iScript™ cDNA Synthesis Kit (Bio-Rad, Cat # 1708890), according to manufacturer’s guidelines. Quantitative gene expression analysis was performed using the CFX96 Touch Real-Time PCR Detection System (Bio-Rad). The following primers for MDGA1, MDGA2, and GAPDH were designed using the Clone Manager 9 software: *MDGA1_Forward: GCCCATGTACCCATTAACCC*; MDGA1_Reverse: *GGACACTCTCCCTTCTTCAG*; MDGA2_Forward: *CAACGTGAAGCCAAGAGAAG*; MDGA2_Reverse: *CAGCACTCGTATTGGATAGG*, GAPDH Forward: *TGAAGCAGGGATCTGAGGG*; GAPDH reverse: *CGAAGGTGGAAGAGTGGGAG*). Each qRT-PCR reaction contained 7.5 μL Power SYBR Green PCR Master Mix (Applied Biosystems, Cat # 4368577), 1.5 μL cDNA, and 1 μL primer master mix (0.1 μM). PCR conditions for all primer sets were are follows: Initial denaturation for 4 min at 95 °C, followed by 41 cycles of 15 s at 95 °C, 20 s at 60 °C, and 30 s at 72 °C. Data analysis was performed using the mean Cycle Quantification (Cq) values of duplicate measurements. Cq values for MDGA1 and MDGA2 were normalized to the respective Cq values for GAPDH, relative expression was calculated using the ΔΔCq method, and final results are expressed as % WT.

### Immunoblotting

Hippocampi from WT and MDGA2 Het mice were dissected, rapidly frozen in liquid nitrogen, and stored at −80 °C. They were homogenized in Syn-PER™ Synaptic Protein Extraction Reagent (Thermo Scientific, Cat # 87793) with protease inhibitors (Thermo scientific Halt™) using a Kimble dounce homogenizer. Total protein concentration was determined using a BCA assay (Pierce, Cat # 23225), and 2.5 µg protein per sample was loaded for SDS-PAGE. Prior to loading, samples were heated to 95 °C in Laemmli buffer (1% SDS, 62.5 mM Tris, 10% glycerol, 1% β-mercaptoethanol, 0.01% bromophenol blue, pH 6.8) for 5 min. Samples were resolved on Mini-PROTEAN® TGX™ Precast Gels (Bio-Rad, Cat # 4561086) and transferred onto trans-blot turbo mini 0.2 µm nitrocellulose transfer packs (Bio-Rad, Cat # 1704158) following the manufacturer guidelines. After protein transfer, the membranes were stained for total protein loading using a Revert™ 700 Total Protein Stain kit (LI-COR, Cat # 926-11016). Subsequently, they were blocked in 3% Milk in 1X TBS containing 0.1% Tween® 20 detergent (TBST) for 1 hr and incubated with anti-MDGA1 antibody (Synaptic Systems, Cat # 421002, 1:1000 dilution in 3% Milk in 1X TBST) at 4 °C overnight. After washing for 3 × 5 min with 1X TBST, the membranes were incubated with a secondary antibody (IRDye® 800CW (LI-COR) Goat anti-Rabbit IgG, 1:10,000 dilution in 3% Milk in 1X TBS) 1 h at room temperature. Blots were washed and scanned on an Odyssey Infrared Imager (LI-COR Biosciences). Signal intensity for each sample was quantified using the image studio Ver 5.2 software. Each sample intensity value was divided by the total protein loading value for the corresponding lane, and normalized to the average sample value of all lanes on the same blot to correct for blot-to-blot variance. Data are expressed as a percentage of the WT average.

### Slice electrophysiology

Young adult mice (6–8 weeks old), arranged in sex-matched sets of four genotypes, were used for experiments within a few days of each other. Mice were anesthetized with Avertin (2,2,2-Tibromoethanol, Sigma) and subsequently transcardially perfused for 100 s with ice-cold artificial cerebrospinal fluid (aCSF) containing (in mM): 64 NaCl, 2.5 KCl, 25 NaHCO_3_, 1.25 NaH_2_PO_4_ and supplemented with (in mM) 7 MgCl_2_, 0.5 CaCl_2_, 10 Glucose, and 120 Sucrose^58^. Mice were decapitated, and brains were rapidly dissected. Two coronal cuts were performed to isolate the hippocampal regions, which were then transferred to a chamber filled with the ice-cold sucrose-aCSF. Subsequently, tissue blocks containing the hippocampal formation were mounted and 300 μM thick coronal sections for sIPSC and mIPSC recordings and approx. 300 μM thick coronal sections for mEPSC were cut on a vibratome (Leica VT1000, Leica, Germany). Slices containing the dorsal hippocampal CA1 were placed in a chamber filled with normal aCSF containing (in mM): 125 NaCl, 2.5 KCl, 25 NaHCO_3_, 1.25 NaH_2_PO_4_, 25 Glucose, 2 CaCl_2_ and 1 MgCl_2_ (continuously bubbled with 95% O_2_ and 5% CO_2_; pH = 7.3, osmolarity = 300 mOsm). Slices were allowed to recover for 30 min at 35 °C and maintained at room temperature (RT) for up to 4.5 h. Chemicals were obtained from Tocris Bioscience (Bristol, Uk) and Sigma Aldrich (Darmstadt, Germany).

Whole-cell patch-clamp recordings were conducted at RT (~22 °C). During recordings, slices were continuously perfused with normal aCSF at a rate of 1.5–2 ml/min. Hippocampal CA1 pyramidal neurons were visually identified using an upright microscope equipped with infrared video microscopy and a 60× objective. Patch pipettes (2.5–4.55 MΩ open tip resistance) were pulled from borosilicate glass (GB150-8P Science Product, Hofheim, Germany). The holding potential was set to −65 mV and spontaneous inhibitory postsynaptic currents (sIPSCs) and miniature inhibitory postsynaptic currents (mIPSC) were recorded under voltage clamp with patch pipettes filled with an internal solution containing in (mM): 135 KCl, 15 K-gluconate, 10 EGTA, 10 HEPES, 2 MgCl_2_, 2 Na_2_-ATP (osmolarity = 332 mOsm). To pharmacologically isolate GABAergic postsynaptic currents, 2 μM 6-cyano-7-nitroquinoxaline-2,3-dione (NBQX, Cat# HB0443, Hello Bio, Bristol, UK) and 2 μM (R)-3-(2-Carboxypiperazin-4-yl)-propyl-1-phosphonic acid ((R)-CPP, Cat#0330, Hello Bio, Bristol, UK) were added to the bath to block AMPARs and NMDARs, respectively. During sIPSC recordings, 2 mM 4N-(2,6-Dimethylphenylcarbamoylmethyl) triethylammonium bromide (QX314, Cat # HB1030, Hello Bio, Bristol, UK) was added to the pipette solution to block voltage-activated Na^+^ currents. During mIPSC recordings, 1 μM tetrodotoxin (TTX, Cat#T-550, Alomone Lab), was added to the bath solution to block voltage-activated Na^+^ currents and suppress action potential (AP) firing. To record miniature excitatory postsynaptic currents (mEPSCs), the holding potential set at -70 mV and recordings were conducted using patch pipettes filled with internal solution containing (in mM) 100 Cs-gluconate, 30 TEA-Cl, 30 CsCl, 10 HEPES, 5 EGTA, 2 Na-phosphocreatine, 4 ATP-Mg, 0.3 GTP (CsOH was used to adjust pH value to 7.2, CsCl was used to adjust osmolarity to 330 mOsm). To pharmacologically isolate mEPSCs, 1 μM tetrodotoxin (TTX, Cat#T-550, Alomone Lab) and 20 μM bicuculline (Cat#0130, Tocris-Cookson, Ellisville, MO) were added to the bath.

Membrane resistance and cell capacitances were estimated from current transients recorded under voltage clamp in response to 10 mV depolarizing voltage steps from a holding potential of −70 mV, and calculated by assuming a simplified two-compartment equivalent circuit model^59^. AP firing threshold was estimated with a ramp protocol under current clamp by injecting depolarizing current increasing from 0 pA up to 100 pA, 200 pA or 300 pA. AP phase-plane plots were constructed from the responses to the lowest depolarization exceeding AP firing threshold. During voltage clamp, a variable fraction of series resistance compensation was applied in order to maintain a residual uncompensated series resistance of 6.25 MΩ. Recordings with an initial uncompensated series resistance of >12.5 MΩ were discarded. The series resistance before compensation was allowed to change by no more than 20% during recordings. Recordings with a leak current >300 pA were discarded. The identity of visually identified CA1 pyramidal cells was confirmed based on their passive membrane properties and their discharge behaviour in response to depolarizing current steps. Patch-clamp data were acquired using an EPC-10 amplifier and Pulse or Patchmaster software (HEKA Elektronik, Germany), using a low-pass Bessel filter with at a cut-off frequency of 5 kHz and digitized at 50 kHz. All offline analyses were performed with IgorPro (Wavemetric, USA). Spontaneously occurring postsynaptic currents (sIPSCs, mIPSCs, mEPSCs) were detected using a sliding template-matching algorithm implemented in IgorPro^60^ after additional offline filtering using a Gaussian low-pass filter with a cut-off frequency of 1 kHz. Amplitudes of individual sIPSCs, mIPSCs and mEPSCs were obtained from the respective template scaling factors. Amplitudes of individual mIPSCs were additionally estimated as the difference between the locally defined baseline and their peak values obtained within appropriately defined time windows. Because template scaling factor-based and peak measurement-based mIPSC amplitude estimates were similar, they were averaged. Rise times of individual mIPSCs were estimated by the steepest slope values obtained from moving linear regressions fitted over a time window of 440 µs corresponding to 22 sampling intervals. Using the individual mIPSC amplitudes, the steepest slope values were subsequently converted to 20-80% rise time values, which assumes a nearly linear rise from 20% to 80% peak amplitude. Additionally, individual mIPSC rise times were estimated from the time points of the respective amplitude intersections during the mIPSC rising phase. Since both rise-time estimates resulted in similar values, they were averaged. The cut-off threshold to distinguish fast mIPSCs putatively arising at proximal inputs from slow IPSCs putatively arising at distal inputs was set to 400 µs^61^. On average, a total of 1596 mIPSCs were analysed for each CA1 pyramidal neuron (minimum 568 events, maximum 2961 events) of which on average 78 events were discarded due to contamination with excessive noise or because of a superposition of multiple events. The fraction of fast mIPSCs (rise times ≤400 µs) was on average 57% for WT recordings.

### Behavioral analysis

Adult mice (8–12 weeks old), arranged in sex-matched sets of four genotypes that were tested on the same day, were assessed for anxiety-related behaviors in an open field test (OF) as previously described^9,46^. The OF was performed in a square arena (50 × 50 cm) made of white plastic, with a 25 × 25 cm center defined during analysis. Mice were placed in one corner and were permitted to explore the arena for 10 min. Performance was recorded using an overhead camera system and scored automatically using the Viewer software (Biobserve, St. Augustin, Germany). Between each mouse, the arena was cleaned thoroughly with 70% ethanol followed by water to eliminate any odors left by the previous mouse.

### Statistical analysis

Statistical analysis was performed using Prism (GraphPad Software, La Jolla, CA, USA) and IgorPro (Wavemetric, USA). Outliers were identified and removed using the Mean and Standard Deviation Method with a threshold value of 2, and data were subjected to two-way ANOVA with Nlgn2 genotype and MDGA genotype as the two factors. Significant main effects of Nlgn2 and/or MDGA genotype, and/or significant Nlgn2 x MDGA interactions, are reported as light grey texts with asterisks above the corresponding graphs, and all effects are reported in Table 1 (Figs. 2–6) or Supplementary Table 7 (Supplementary Fig. 3/4). Post-hoc analysis was conducted using Tukey´s test for multiple comparisons between groups, and significant differences are indicated with asterisks above the corresponding comparison. Unpaired *t*-tests were used to compare parameters obtained from the comparison of WT and MDGA1 KO mice in Fig. 5 where differences were not statistically significant. All effects are reported in Supplementary Tables 5 and 6 (Fig. 5).

### Reporting summary

Further information on research design is available in the Nature Portfolio Reporting Summary linked to this article.

## Supplementary information

Supplementary information

Description of Additional Supplementary Materials

Supplementary Data 1

Supplementary Data 2

Reporting summary

## Data Availability

All source data are available online in the files Supplementary Data 1 (main figures) and Supplementary Data 2 (Supplementary Figs.). Further information is available from the corresponding author upon request.
