# Supplementary material for: Functional Neuroligin-2-MDGA1 interactions differentially regulate synaptic GABAARs and cytosolic gephyrin aggregation
Source: Commun Biol. 2024 Sep 17;7:1157. doi: 10.1038/s42003-024-06789-z (PMC11405390; doi:10.1038/s42003-024-06789-z)
Supplement: Supplementary file 1 — Supplementary information [file 42003_2024_6789_MOESM1_ESM.pdf]

SUPPLEMENTARY INFORMATION

Functional Neuroligin-2-MDGA1 interactions differentially regulate synaptic $GABA_A$Rs and cytosolic gephyrin aggregation

Tommaso Zeppillo[#], Heba Ali[#], Sowbarnika Ravichandran[§], Tamara C. Ritter[§], Sally Wenger, Francisco J. López-Murcia, Erinn Gideons, Janetti Signorelli, Michael J. Schmeisser, Jens Wiltfang, JeongSeop Rhee, Nils Brose, Holger Taschenberger and Dilja Krueger-Burg

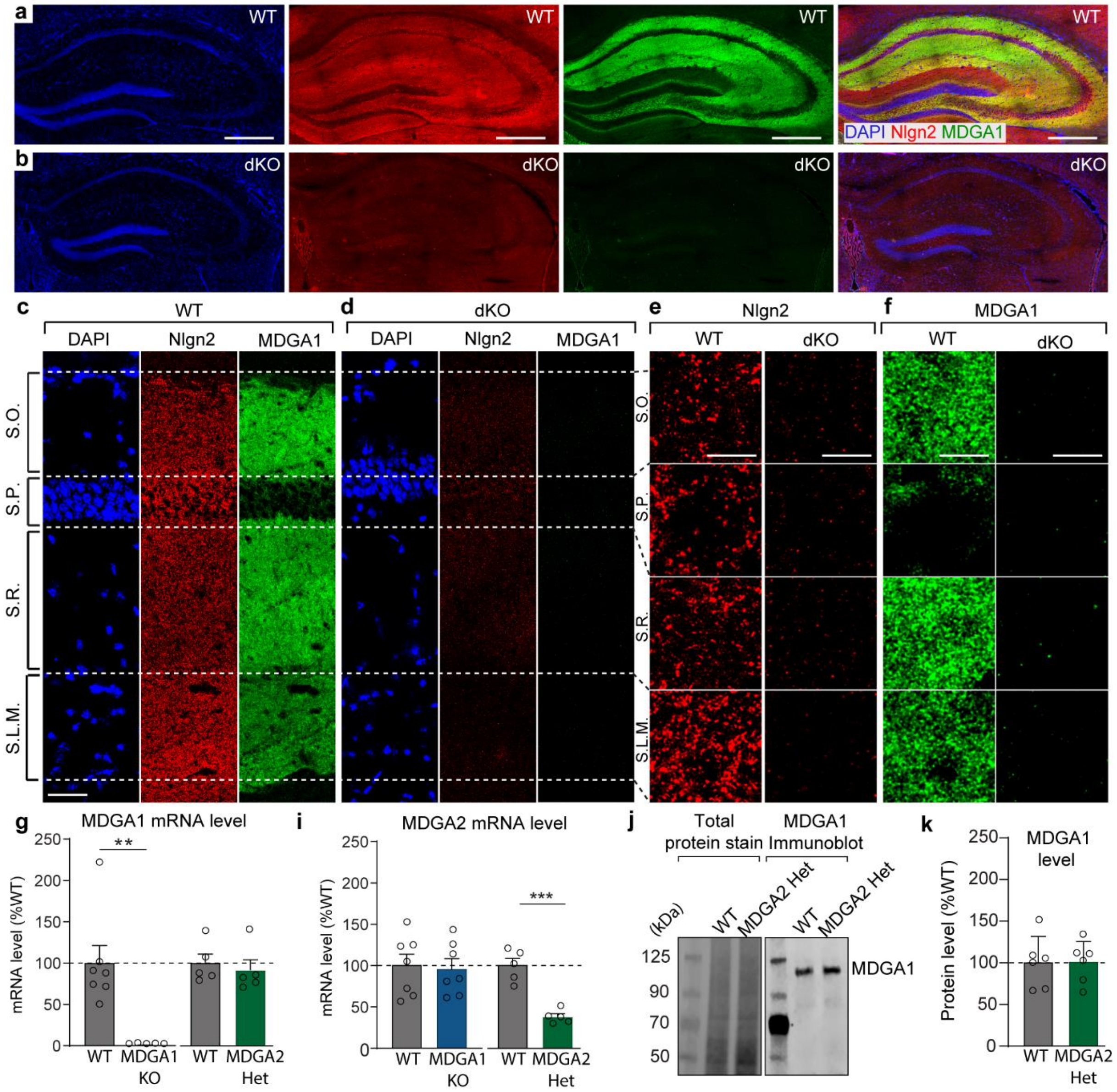

Supplementary Fig. 1: Validation of the specificity of antibodies against MDGA1 and Nlgn2, and of the mouse models used in the study. (a-b) Photomicrographs showing an overview of the hippocampus in WT (a) versus Nlgn2-MDGA1 dKO mice (b) labelled with DAPI (blue), and antibodies against Nlgn2 (red) and MDGA1 (green). Scale bar 500 µm. **(c-d)** Photomicrographs showing an overview of area CA1 labelled with DAPI, and with antibodies against Nlgn2 and MDGA1 in WT (c) versus Nlgn2 / MDGA1 dKO mice (d). Scale bar 50 µm. **(e-f)** High magnification photomicrographs showing Nlgn2 and MDGA1 labeling within different hippocampal layers in WT (e) versus Nlgn2 / MDGA1 dKO mice (f). Scale bar 5 µm. (**g**)**.** Bar graph showing MDGA1 mRNA level in WT, MDGA1 KO and MDGA1 Het mice, relative values normalized by the expression of the glyceraldehyde-3-phosphate dehydrogenase (GAPDH) and expressed as percentage of the WT mice. **(i)** Bar graph showing the MDGA2 mRNA level in WT, MDGA1 KO and MDGA1 Het mice. **(j)** Western blot membrane showing the total protein stain and the result of the immunoblot against MDGA1 in WT and MDGA2 Het mice. (**k**) Bars graph showing the MDGA1 protein level between WT and MDGA2 Het mice normalized by the average sample value of all lanes on the same blot, and expressed as a percentage of the WT mice. Statistically significant unpaired t-test: * $p<0.05$, ** $p<0.01$, *** $p<0.001$. Error bars represent SEM, and each circle represents an experimental animal (n = 5-7), details listed in supplementary table 1.

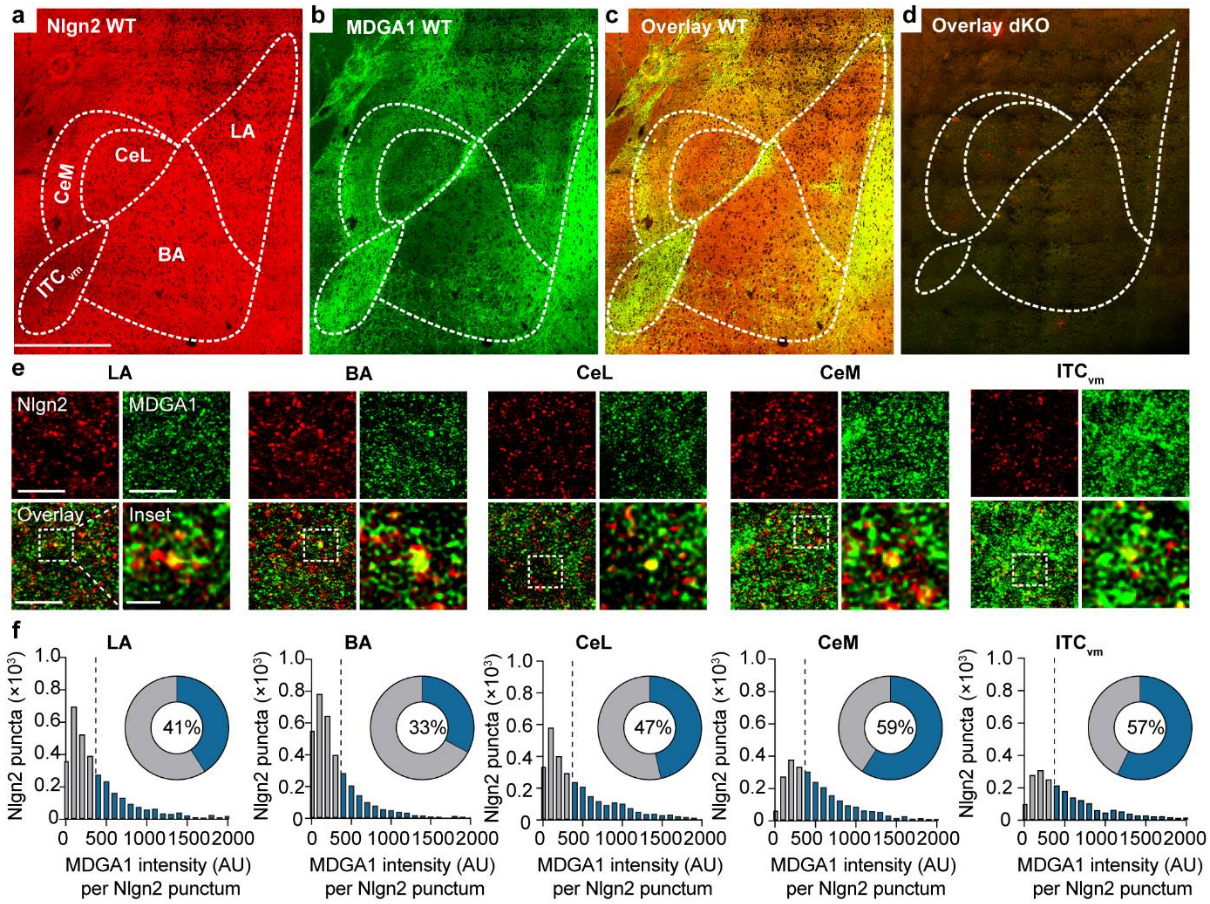

Supplementary Fig. 2. Colocalization of Nlgn2 and MDGA1 in the amygdala of WT mice. (a-d) Photomicrographs showing the amygdala in WT (a-c) and dKO (d) mice, labeled with antibodies against Nlgn2 (a), MDGA1 (b), or an overlay of both antibodies (c, d). (e) High magnification photomicrographs showing representative images of Nlgn2 (red) and MDGA1 (green) staining, and their colocalization (overlay) in subregions of the amygdala. LA, lateral amygdala; BA basal, amygdala; CeL, centrolateral amygdala; CeM, centromedial amygdala; ITC_{vm}, ventromedial intercalated cluster. **(f)** Histograms showing the frequency distribution of MDGA1 fluorescence (intensity in arbitrary units) within Nlgn2-labeled puncta in subregions of the amygdala (LA, BA, CeL, CeM, ITC_{vm}). Bars in blue represent Nlgn2-labeled puncta with above-threshold MDGA1 fluorescence intensity (see Methods section for threshold determination). Doughnut chart insets display the percentage of Nlgn2-labelled puncta with an above-threshold MDGA1 fluorescence intensity (in blue, percentage in the center of the doughnut chart).

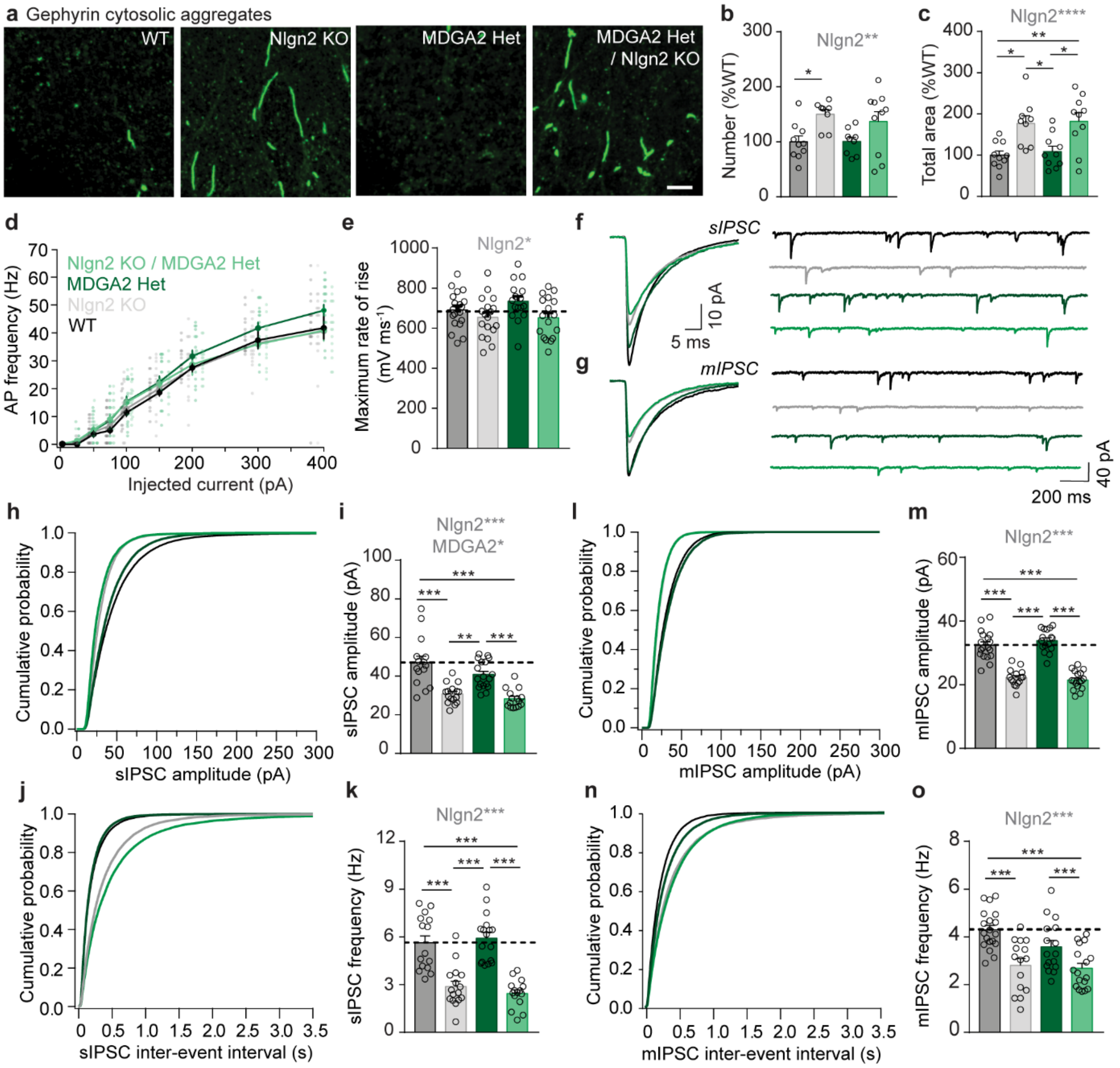

Supplementary Fig. 3: Heterozygous MDGA2 deletion does not affect the formation of gephyrin aggregates nor GABAergic transmission in CA1 pyramidal cells. (a) High magnification photomicrographs of gephyrin aggregates in the hippocampal CA1 area of WT, Nlgn2 KO, MDGA2 Het and Nlgn2 KO / MDGA2 Het mice. Scale bar 5 µm. **(b-c)** Quantification of the number (b) and the total area (c) of gephyrin aggregates, expressed as percentage of WT. Statistically significant ANOVA comparisons are marked in gray at the top of panels and listed in Supplementary Table 3. For all other ANOVA comparisons, F<1. Post-hoc analysis (Tukey's comparison test): * p<0.05, ** p<0.01, *** p<0.001. Error bars represent SEM, and each circle represents an experimental animal (n = 8-10). **(d)** Frequency of action potentials (APs) in response to depolarizing current steps. **(e)** Quantification of the maximal rate of AP rise in CA1 pyramidal neurons of WT, Nlgn2 KO, MDGA2 Het, Nlgn2 KO / MDGA2 Het mice. **(f)** Representative average sIPSC waveforms (left) obtained from individual sIPSCs (right) recorded in the four genotypes. **(g)** Representative average mIPSC waveforms (right) obtained from individual mIPSCs (right) recorded in the four genotypes. **(h-k)** Average cumulative distributions of sIPSC amplitudes (h) and sIPSC inter-event intervals (j) shown together with the respective mean values for sIPSC amplitudes (i) and sIPSC frequencies (k) for all genotypes. **(l-o)** Average cumulative distributions of mIPSC amplitudes (m) and mIPSC inter-event intervals (n) shown together with the respective mean values for mIPSC amplitudes (i) and mIPSC frequencies (o) for all genotypes. Statistically significant ANOVA comparisons are marked in gray at the top of panels and listed in Supplementary Table 8. For all other ANOVA comparisons, F<1. Post-hoc analysis (Tukey's comparison test): * p<0.05, ** p<0.01, *** p<0.001,. Error bars represent SEM, and each circle represents a single cell (n = 14-19 cells for APs and rate of rise; 16-18 cells for sIPSC recordings; 15-19 cells for mIPSC recordings; four animals per genotype).

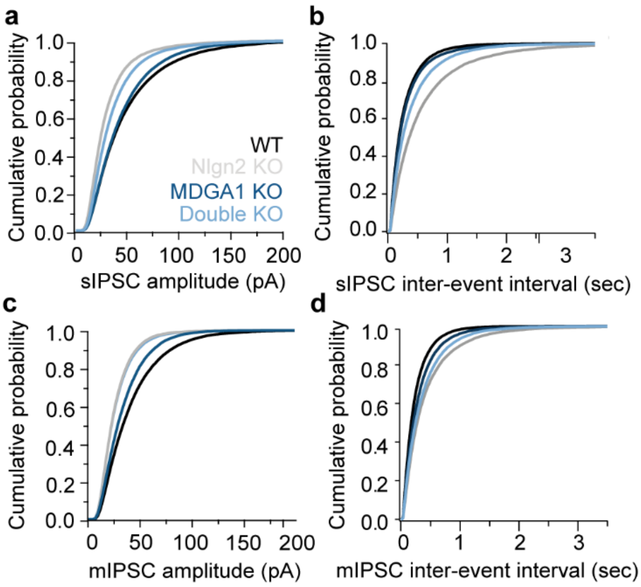

Supplementary Fig. 4: Loss of MDGA1 expression perturbs spontaneous GABAergic transmission in CA1 pyramidal neurons. (a-b) Average cumulative amplitude distributions and average waveforms of sIPSCs amplitude (a) and frequency (b) for all genotypes (WT, Nlgn2 KO, MDGA1 KO, and Nlgn2-MDGA1 double KO). **(c-d).** Average cumulative distributions of mIPSCs amplitude (c) and frequency **(d)** for all analyzed genotypes.

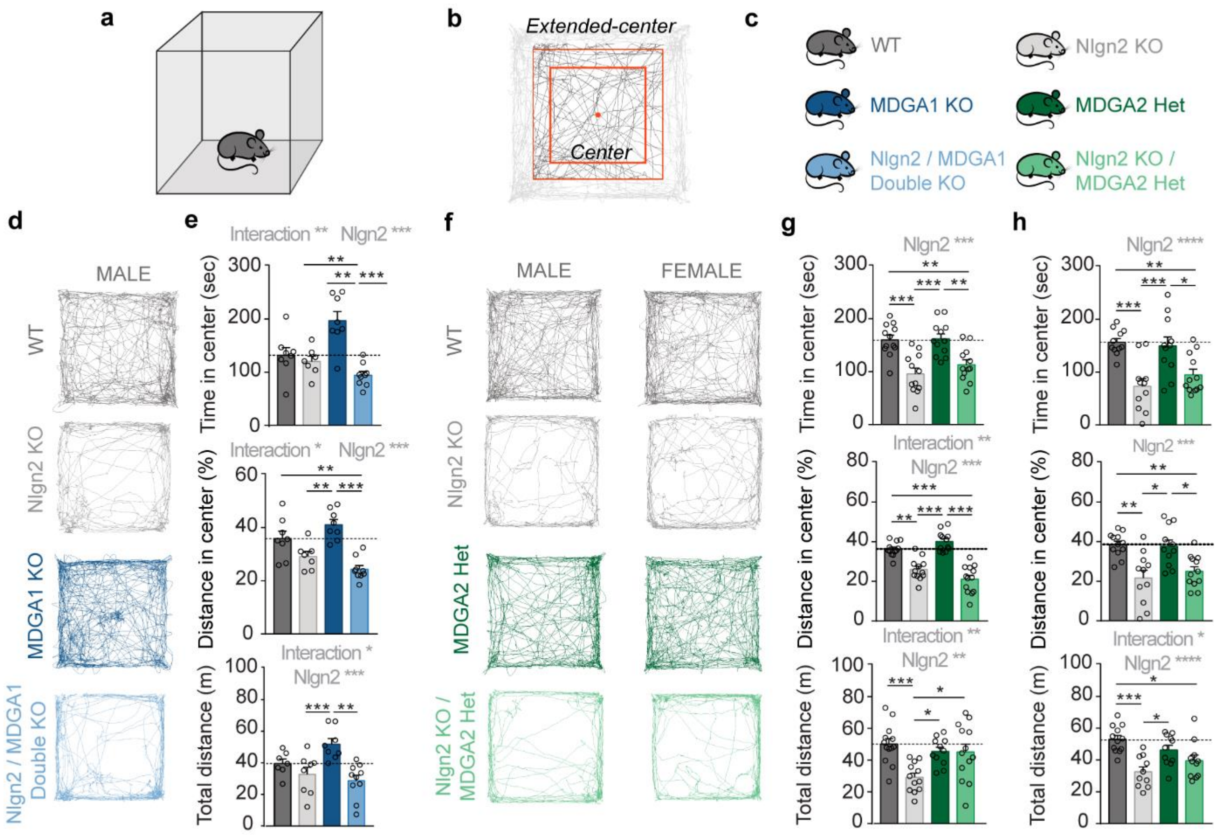

Supplementary Fig. 5. Heterozygous MDGA2 deletion does not influence abnormal anxiety-related avoidance behavior in Nlgn2 KO mice. (a-c) Schematics representing the OF arena (a), the center (b), and the genotypes analyzed (c). **(d)** Representative tracks of OF exploration in MDGA1 male mice. **(e)** OF scores of MDGA1 male mice: Time spent in the anxiogenic region (top) of the OF arena, distance traveled in the center of the OF expressed as percentage of total distance traveled (center), total distance travelled in the OF (bottom). **(f)** Representative tracks of OF exploration in MDGA2 mice. **(g)** OF scores of MDGA2 male mice: Time spent in the anxiogenic region (top) of the OF arena, distance traveled in the center of the OF expressed as percentage of total distance traveled (center), total distance travelled in the OF (bottom). **(h)** OF scores of MDGA2 female mice: Time spent in the anxiogenic region (top) of the OF arena, distance traveled in the center of the OF expressed as percentage of total distance traveled (center), total distance travelled in the OF (bottom). Statistically significant ANOVA comparisons are marked in gray at the top of panels and listed in Supplementary Table 8. For all other ANOVA comparisons, F<1. Post-hoc analysis (Tukey's comparison test): * $p<0.05$, ** $p<0.01$, *** $p<0.001$. Error bars represent SEM, and each circle represents an experimental animal (n = 7-9 for male MDGA1 set, n = 11-13 for female MDGA2 set, n = 10-12 for male MDGA2 set).

Supplementary Table 1. Summary of MDGA1 and MDGA2 mRNA and protein levels. MDGA1 and MDGA2 mRNA levels in hippocampal tissue of WT, MDGA1 KO and MDGA2 Het mice (unpaired t-test) were normalized by the expression of the glyceraldehyde-3-phosphate dehydrogenase (GAPDH) mRNA, and expressed as percentage of the expression in WT mice. MDGA1 protein level between WT and MDGA2 Het mice were normalized by the average sample value of all lanes on the same blot, and expressed as a percentage of WT mice. (Avg.Cq represent the cycle number at which the sample's reaction curve intersects the threshold line).

	**WT**		**MDGA1 KO**		
	**n**	**Mean ± SEM**	**n**	**Mean ± SEM**	**p-value**
**MDGA1 mRNA level (S1g)**	7	100.0 ± 21.3	5	0.6 ± 0.2	<0.001
**MDGA2 mRNA level (S1i)**	7	100.0 ± 11.0	7	91.6 ± 12.7	0.631
**Avg.Cq MDGA1**	7	31.2 ± 0.3	5	38.6 ± 0.4	<0.001
**Avg.Cq MDGA2**	7	30.6 ± 0.2	7	30.9 ± 0.3	0.515
**Avg.Cq GAPDH**	7	23.2 ± 0.3	5	23.1 ± 0.3	0.849
	WT		***MDGA2 Het***		
	n	***Mean ± SEM***	***n***	***Mean ± SEM***	***p-value***
MDGA1 mRNA level (S1i)	5	100.0 ± 11.0	5	91.6 ± 12.7	0.631
MDGA2 mRNA level (S1i)	5	100.0 ± 8.6	5	39.6 ± 3.8	<0.001
Avg.Cq MDGA1	5	31.6 ± 0.2	5	31.4 ± 0.2	0.516
Avg.Cq MDGA2	5	30.1 ± 0.2	5	31.1 ± 0.1	0.003
Avg.Cq GAPDH	5	23.2 ± 0.1	5	22.88 ± 0.17	0.166
MDGA1 Protein expression (Immunoblotting) – S1j-k	5	100.0 ± 12.7	5	100.4 ± 10.1	0.983

Supplementary Table 2. Analysis of the number and size of gephyrin, GABA$_A$Rγ2 and VIAAT puncta in layers S.O., S.P., S.R. and S.L.M. of hippocampal area CA1 in WT, Nlgn2 KO, MDGA1 KO and Nlgn2 / MDGA1 dKO mice (all data expressed as percentage of WT).

		**WT**		**Nlgn2 KO**		**MDGA1 KO**		**Nlgn2 / MDGA1 dKO**		**Main source of variation**	
		n	Mean ± SEM	n	Mean ± SEM	n	Mean ± SEM	n	Mean ± SEM	F-value	p-value
**Stratum oriens (S.O.)**	Gephyrin (number)	8	100.0 ± 6.4	8	79.0 ± 7.0	8	86.6 ± 5.7	8	76.6 ± 9.6	Nlgn2: $F_{(1,28)} = 4.48$	0.04
	Gephyrin (size)	8	100.0 ± 3.2	8	90.3 ± 3.3	7	94.2 ± 2.2	7	91.3 ± 3.4	Nlgn2: $F_{(1,28)} = 4.23$	0.05
	GABA$_A$Rγ2 (number)	8	100.0 ± 5.3	8	90.3 ± 6.3	9	93.1 ± 7.8	9	3.0 ± 6.5	/	/
	GABA$_A$Rγ2 (size)	9	100.0 ± 3.9	8	81.5 ± 4.1	9	84.9 ± 3.5	9	82.0 ± 3.3	Nlgn2: $F_{(1,31)} = 8.38$<br>Interaction $F_{(1,31)} = 4.48$	Nlgn2: 0.01<br>Interaction 0.04
	VIAAT (number)	8	100.0 ± 15.7	8	101.2 ± 12.1	8	87.1 ± 11.9	8	79.7 ± 14.1	/	/
	VIAAT (size)	8	100.0 ± 2.4	8	99.0 ± 3.2	8	89.0 ± 3.1	8	87.6 ± 1.9	MDGA1: $F_{(1,28)} = 18.40$	<0.001
**Stratum lacunosum moleculare (SLM)**	Gephyrin (number)	7	100.0 ± 7.9	7	98.5 ± 8.7	7	109.9 ± 4.4	6	93.2 ± 2.8	/	/
	Gephyrin (size)	8	100.0 ± 3.8	8	95.6 ± 2.3	7	98.0 ± 1.8	8	91.9 ± 4.3	/	/
	GABA$_A$Rγ2 (number)	8	100.0 ± 6.0	8	94.6 ± 4.1	8	107.7 ± 4.0	9	102.6 ± 4.4		
	GABA$_A$Rγ2 (size)	8	100.0 ± 4.3	9	85.5 ± 4.1	8	84.2 ± 2.7	9	83.2 ± 3.4	Nlgn2: $F_{(1,30)} = 4.38$<br>MDGA1: $F_{(1,30)} = 5.94$	Nlgn2: 0.05<br>MDGA1: 0.02
	VIAAT (number)	6	100.0 ± 26.4	6	10.6 ± 22.3	4	117.5 ± 23.8	4	120.3 ± 17.9	/	/
	VIAAT (size)	7	100.0 ± 3.3	6	100.8 ± 5.5	5	85.2 ± 3.0	5	94.0 ± 3.2	MDGA1: $F_{(1,19)} = 7.10$	MDGA1: 0.02

Supplementary Table 3 (Part 1). Analysis of the number and size of gephyrin, GABA$_A$Rγ2 and VIAAT puncta in layers S.O., S.P., S.R. and S.L.M. of hippocampal area CA1 in WT, Nlgn2 KO, MDGA2 Het and Nlgn2 KO / MDGA2 Het mice (all data expressed as percentage of WT)

		WT		Nlgn2 KO		MDGA2 Het		Nlgn2 KO / MDGA2 Het		Main source of variation	
		n	Mean ± SEM	n	Mean ± SEM	n	Mean ± SEM	n	Mean ± SEM	F-value	p-value
Stratum oriens (S.O.)	Gephyrin (number)	11	100.0 ± 8.9	10	86.9 ± 6.5	11	104.3 ± 8.6	9	78.3 ± 7.0	Nlgn2: $F_{(1,37)} = 6.0$	Nlgn2: 0.02
	Gephyrin (size)	10	100.0 ± 3.0	10	91.4 ± 2.4	10	96.7 ± 2.8	11	94.1 ± 2.3	Nlgn2: $F_{(1,37)} = 4.5$	Nlgn2: 0.04
	GABA$_A$Rγ2 (number)	12	100.0 ± 7.7	11	87.6 ± 8.9	12	117.9 ± 13.8	12	60.3 ± 8.1	Interaction: $F_{(1,43)} = 5.1$ Nlgn2: $F_{(1,43)} = 12.3$	Interaction: 0.03 Nlgn2: 0.001
	GABA$_A$Rγ2 (size)	10	100.00 ± 2.5	10	86.2 ± 3.1	12	93.8 ± 5.0	11	76.8 ± 2.2	Nlgn2: $F_{(1,39)} = 18.65$ MDGA2: $F_{(1,39)} = 4.7$	Nlgn2: <0.001 MDGA2: 0.04
	VIAAT (number)	7	100.0 ± 17.7	7	85.0 ± 11.0	6	83.0 ± 18.4	6	58.6 ± 9.6	/	/
	VIAAT (size)	7	100.0 ± 5.3	7	99.3 ± 3.1	6	90.0 ± 2.5	7	93.2 ± 6.1	/	/
Stratum pyramidale (S.P.)	Gephyrin (number)	9	100.0 ± 10.7	9	88.7 ± 6.9	9	79.6 ± 7.1	9	70.1 ± 8.7	MDGA2: $F_{(1,32)} = 5.3$	MDGA2: 0.03
	Gephyrin (size)	9	100.0 ± 2.4	9	90.3 ± 3.6	9	92.7 ± 3.1	8	89.2 ± 1.3	Nlgn2: $F_{(1,31)} = 5.5$	Nlgn2: 0.03
	GABA$_A$Rγ2 (number)	9	100.0 ± 4.0	10	81.3 ± 7.6	10	84.8 ± 8.7	9	59.5 ± 5.2	Nlgn2: $F_{(1,34)} = 10.4$ MDGA2: $F_{(1,34)} = 7.4$	Nlgn2: 0.003 MDGA2: 0.01
	GABA$_A$Rγ2 (size)	10	100.0 ± 5.4	10	88.9 ± 4.6	10	87.3 ± 3.9	10	70.8 ± 3.4	Nlgn2: $F_{(1,36)} = 10.0$ MDGA2: $F_{(1,36)} = 12.3$	Nlgn2: 0.003 MDGA2: 0.001
	VIAAT (number)	8	100.0 ± 19.3	8	91.4 ± 10.9	8	71.3 ± 11.8	7	86.3 ± 12.5	/	/
	VIAAT (size)	8	100.0 ± 9.2	8	98.1 ± 3.0	7	109.3 ± 3.7	7	103.7 ± 4.7	/	/
Stratum radiatum (S.R.)	Gephyrin (number)	11	100.0 ± 10.7	9	85.6 ± 8.2	11	94.9 ± 9.1	8	92.6 ± 3.6	/	/
	Gephyrin ‘ (size)	10	100.0 ± 3.3	9	95.7 ± 1.8	9	94.9 ± 2.5	10	93.6 ± 2.3	/	/
	GABA$_A$Rγ2 (number)	11	100.0 ± 8.0	11	75.0 ± 9.2	12	90.9 ± 9.1	12	56.0 ± 8.7	Nlgn2: $F_{(1\ 42)} = 11.48$	Nlgn2: 0.002
	GABA$_A$Rγ2 (size)	10	100.0 ± 3.4	10	83.9 ± 3.0	11	100.0 ± 5.2	11	78.2 ± 3.0	Nlgn2: $F_{(1,38)} = 24.41$	Nlgn2: <0.001
	VIAAT (number)	7	100.0 ± 25.2	7	81.2 ± 13.2	7	68.7 ± 11.5	5	90.4 ± 7.1	/	/
	VIAAT (size)	7	100.0 ±7.1	7	101.76 ± 2.8	7	92.5 ± 4.6	7	99.5 ± 5.0	/	/

Supplementary Table 3 (Part 2). Analysis of the number and size of gephyrin, $GABA_AR\gamma2$ and VIAAT puncta in layers S.O., S.P., S.R. and S.L.M. of hippocampal area CA1 in WT, Nlgn2 KO, MDGA2 Het and Nlgn2 KO / MDGA2 Het mice (all data expressed as percentage of WT).

		WT		Nlgn2 KO		MDGA2 Het		Nlgn2 KO / MDGA2 Het		Main source of variation	
		n	Mean ± SEM	n	Mean ± SEM	n	Mean ± SEM	n	Mean ± SEM	F-value	p-value
Stratum lacunosum moleculare (S.L.M.)	Gephyrin (number)	10	100 .0 ±11.2	9	99.2 ± 3.6	11	88.8 ± 5.4	8	108.4 ± 6.4	/	/
	Gephyrin (size)	11	100.0 ± 3.6	9	94.4 ± 1.3	11	93.5 ± 2.6	10	92.6 ± 1.9	/	/
	$GABA_AR\gamma2$ (number)	9	100.0 ± 8.9	7	85.9 ± 3.4	10	82.2 ± 9.8	9	71.6 ± 10.0	/	/
	$GABA_AR\gamma2$ (size)	10	100.0 ± 4.8	9	84.3 ± 2.7	9	88.3 ± 3.2	9	78.0 ±1.7	Nlgn2: $F_{(1,33)} = 14.5$ MDGA2: $F_{(1,33)} = 6.9$	Nlgn2: <0.001 MDGA2: 0.01
	VIAAT (number)	5	100.0 ± 20.6	6	49.1 ± 11.2	8	30.5 ± 8.5	7	32.9 ± 6.2	Interaction: $F_{(1,22)} = 5.5$ Nlgn2: $F_{(1,22)} = 4.6$ MDGA2: $F_{(1,22)} = 14.2$	Interaction: 0.03 Nlgn2: 0.04 MDGA2: 0.001
	VIAAT (size)	7	100.0 ± 7.8	6	101.1 ± 10.6	8	87.6 ± 7.3	7	99.6 ± 3.7	/	/

Supplementary Table 4. Passive and AP properties of CA1 pyramidal cells in WT, Nlgn2 KO, MDGA1 KO, Nlgn2 KO / MDGA1 double KO, and MDGA2 Het and Nlgn2 KO / MDGA2 Het mice.

	WT		Nlgn2 KO		MDGA1 KO		Nlgn2 / MDGA1 dKO		Main source of variation	
	n	Mean ± SEM	n	Mean ± SEM	n	Mean ± SEM	n	Mean ± SEM	F-value	p-value
**Membrane resistance (MΩ)**	37	100.5 ± 4.5	34	95.0 ± 5.6	41	114.6 ± 7.4	36	100.9 ± 7.7	\	\
**Membrane capacitance, proximal compartments (pF)**	37	42.9 ± 2.4	34	45.1 ± 2.7	41	39.5 ± 1.5	36	46.9 ± 1.8	Nlgn2 KO: $F_{(1,144)} = 5.1$	0.03
**Membrane capacitance, distal compartments (pF)**	37	122.7 ± 4.8	34	113.2 ± 6.0	41	111.2 ± 3.4	36	126.6 ± 5.1	Interaction: $F_{(1,144)} = 6.6$	0.01
**Resting membrane potential (mV)**	21	-58.3 ± 1.7	16	-57.2 ± 2.2	23	-55.4 ± 1.3	18	-59.9 ± 1.5	\	\
**AP threshold (mV)**	20	-44.4 ± 0.8	15	-43.7 ± 0.7	24	-45.4 ± 0.7	18	-44.7 ± 0.7	\	\
**AP amplitude (mV)**	20	117.9 ± 1.2	15	117.8 ± 1.5	24	120.8 ± 1.8	19	120.9 ± 1.4	\	\
**AP maximum rate of rise (mV/ms)**	21	582.4 ± 17.5	16	591.9 ± 34.9	19	648.6 ± 20.2	24	733.8 ± 21.5	Nlgn2 KO: $F_{(1,73)} = 5.3$ Interaction: $F_{(1,73)} = 3.55$	Nlgn2 KO: 0.02 Interaction: 0.004

	WT		Nlgn2 KO		MDGA2 Het		Nlgn2 KO / MDGA2 Het		Main source of variation	
	n	Mean ± SEM	n	Mean ± SEM	n	Mean ± SEM	n	Mean ± SEM	F-value	p-value
**Membrane resistance (MΩ)**	41	92.1 ± 4.4	35	97.4 ± 3.1	36	99.6 ± 5.3	35	104.2 ± 4.6	\	\
**Membrane capacitance, proximal compartments (pF)**	41	45.7 ± 1.9	35	45.5 ± 2.0	36	40.5 ± 1.4	35	42.7 ± 2.3	MDGA2: $F_{(1,143)} = 4.1$	0.04
**Membrane capacitance, distal compartments (pF)**	41	116.4 ± 5.0	35	106.5 ± 3.8	36	107.6 ± 5.6	35	109.6 ± 4.9	\	\
**Resting membrane potential (mV)**	20	-58.8 ± 1.2	19	-60.4 ± 1.5	17	-57.0 ± 1.8	17	-55.9 ± 1.9	MDGA2: $F_{(1,69)} = 4.5$	0.04
**AP threshold (mV)**	19	-45.6 ± 1.0	18	-45.5 ± 0.8	17	-45.6 ± 0.8	17	-45.7 ± 0.6	\	\
**AP amplitude (mV)**	19	117.2 ± 1.0	18	119.1 ± 1.5	17	120.3 ± 1.1	17	116.9 ± 1.5	Interaction: $F_{(1,67)} = 4.6$	0.04
**AP Maximum rate of rise (mV/ms)**	19	690.7 ± 21.0	18	654.4 ± 24.0	16	734.2 ± 24.5	17	652.4 ± 25.0	Nlgn2: $F_{(1,66)} = 6.3$	0.02

Supplementary Table 5. Analysis of the number and size of PSD95 and vGlut puncta in layers S.O., S.P., S.R. and S.L.M. of hippocampal area CA1 in WT and MDGA1 KO mice (all data expressed as percentage of WT).

		WT		Mdga1 KO		
		n	Mean ± SEM	n	Mean ± SEM	p-value
Stratum Oriens (S.O.)	**PSD95 (number)**	6	100.0 ± 7.9	6	82.5 ± 7.9	0.15
	**PSD95 (size)**	6	100.0 ± 2.9	6	100.1 ± 2.9	0.99
	**vGluT1 (number)**	6	100.0 ± 11.6	6	123.7 ± 11.6	0.18
	**vGluT1 (size)**	6	100.0 ± 4.2	6	115.3 ± 4.2	0.03
Stratum Pyramidale (S.P.)	**PSD95 (number)**	6	100.0 ± 11.0	6	70.0 ± 11.0	0.08
	**PSD95 (size)**	6	100.0 ± 3.3	6	99.7 ± 3.3	0.94
	**vGluT1 (number)**	6	100.0 ± 21.8	6	160.8 ± 21.8	0.08
	**vGluT1 (size)**	6	100.0 ± 5.5	6	103.2 ± 5.5	0.68
Stratum Radiatum (S.R.)	**PSD95 (number)**	6	100.0 ± 15.2	6	100.4 ± 15.2	0.99
	**PSD95 (size)**	6	100.0 ± 5.3	6	105.7 ± 5.3	0.47
	**vGluT1 (number)**	6	100.0 ± 15.6	6	123.3 ± 15.6	0.31
	**vGluT1 (size)**	6	100.0 ± 9.8	6	127.8 ± 9.8	0.07
Stratum Lacunoso moleculare (S.L.M.)	**PSD95 (number)**	6	100.0 ± 9.3	6	89.2 ± 9.3	0.43
	**PSD95 (size)**	6	100.0 ± 2.9	6	93.8 ± 2.9	0.17
	**vGluT1 (number)**	6	100.0 ± 12.2	6	85.0 ± 12.2	0.41
	**vGluT1 (size)**	6	100.0 ± 4.5	6	93.2 ± 4.5	0.31

Supplementary Table 6. Comparison of: mean amplitudes and mean frequencies of spontaneous mEPSCs, and passive properties of CA1 pyramidal cells in WT and MDGA1 KO mice (unpaired t-test).

	WT		MDGA1 KO		
	n	Mean ± SEM	n	Mean ± SEM	p-value
**mEPSC frequency**	23	0.12 ± 0.01	22	0.13 ± 0.01	0.07
**mEPSC amplitude**	23	9.4 ± 0.2	22	9.7 ± 0.3	0.52
**Membrane resistance (MOhm)**	23	164.8 ± 6.4	23	149.8 ± 6.0	0.1
**Membrane capacitance, proximal compartments (pF)**	23	34.3 ± 2.5	23	27.2 ± 0.8	0.02
**Membrane capacitance, distal compartments (pF)**	23	135.8 ± 5.9	23	117.4 ± 4.6	0.01

Supplementary Table 7. Two-way ANOVA comparisons for Supplementary Fig. 3/5.

Figure	Nlgn2 x MDGA2 interaction		Main effect of Nlgn2		Main effect of MDGA2	
	F-value	p-value	F-value	p-value	F-value	p-value
**S3b**	$F_{(1,35)} < 1$	0.9	$F_{(1,35)} = 21.5$	<0.001	$F_{(1, 5)} < 1$	0.7
**S3c**	$F_{(1,33)} < 1$	0.6	$F_{(1,33)} = 11.7$	0.002	$F_{(1,33)} < 1$	0.6
**S3e**	$F_{(1,66)} < 1$	0.3	$F_{(1,66)} = 6.3$	0.02	$F_{(1,66)} < 1$	0.4
**S3i**	$F_{(1,59)} = 1.1$	0.3	$F_{(1,59)} = 78.98$	<0.001	$F_{(1,59)} < 1$	0.8
**S3m**	$F_{(1,60)} < 1$	0.4	$F_{(1,60)} = 51.1$	<0.001	$F_{(1,60)} = 4.7$	0.04
**S3k**	$F_{(1,62)} = 1.66$	0.2	$F_{(1,62)} = 171.6$	<0.001	$F_{(1,62)} < 1$	0.7
**S3o**	$F_{(1,63)} = 1.59$	0.2	$F_{(1,63)} = 26.1$	<0.001	$F_{(1,63)} = 3.2$	0.1
**S5g** Time in center	$F_{(1,44)} < 1$	0.8	$F_{(1,44)} = 11.6$	0.001	$F_{(1,44)} = 1.8$	0.2
**S5g** Center distance	$F_{(1,44)} = 5.68$	0.02	$F_{(1,44)} = 56.8$	<0.001	$F_{(1,44)} < 1$	0.4
**S5g** Total distance	$F_{(1\ 45)} = 7.95$	0.01	$F_{(1,45)} = 8.5$	0.01	$F_{(1,45)} = 2.3$	0.1
**S5h** Time in center	$F_{(1,41)} = 1.4$	0.3	$F_{(1,41)} = 13.2$	<0.001	$F_{(1,41)} < 1$	0.7
**S5h** Center distance	$F_{(1,41)} < 1$	1.0	$F(_{1,\ 41)} = 20.4$	<0.001	$F_{(1,41)} < 1$	0.8
**S5h** Total distance	$F_{(1,40)} = 5.19$	0.03	$F_{(1,40)} = 20.7$	<0.001	$F_{(1,40)} < 1$	1.0
**Figure**	**Nlgn2 x MDGA1 interaction**		**Main effect of Nlgn2**		**Main effect of MDGA1**	
	**F-value**	**p-value**	**F-value**	**p-value**	**F-value**	**p-value**
**S5e** Time in center	$F_{(1,27)} = 2.47$	0.13	$F_{(1,27)} = 21.6$	<0.001	$F_{(1,27)} = 3.3$	0.1
**S5e** Center distance	$F_{(1,29)} = 2.8$	0.1	$F_{(1,29)} = 28.6$	<0.001	$F_{(1,29)} < 1$	0.8
**S5e** Total distance	$F_{(1,29)} = 4.67$	0.04	$F_{(1,29)} = 16.0$	<0.001	$F_{(1,29)} = 1.2$	0.3
