# Supplementary material for: Functional Neuroligin-2-MDGA1 interactions differentially regulate synaptic GABAARs and cytosolic gephyrin aggregation
Source: Commun Biol. 2024 Sep 17;7:1157. doi: 10.1038/s42003-024-06789-z (PMC11405390; doi:10.1038/s42003-024-06789-z)
Supplement: Supplementary file 2 — Description of Additional Supplementary Materials [file 42003_2024_6789_MOESM2_ESM.pdf]

Description of Additional Supplementary Files

File name: Supplementary Data 1

Description: Source data for Figures 1-6

File name: Supplementary Data 2

Description: Source data for Supplementary Figures 1-5 and Supplementary Tables 1-6
